# FunSPU: A versatile and adaptive multiple functional annotation-based association test of whole-genome sequencing data

**DOI:** 10.1371/journal.pgen.1008081

**Published:** 2019-04-29

**Authors:** Yiding Ma, Peng Wei

**Affiliations:** 1 Department of Biostatistics, The University of Texas MD Anderson Cancer Center, Houston, Texas, United States of America; 2 Department of Biostatistics and Data Science, School of Public Health, The University of Texas Health Science Center, Houston, Texas, United States of America; Case Western Reserve University, UNITED STATES

## Abstract

Despite ongoing large-scale population-based whole-genome sequencing (WGS) projects such as the NIH NHLBI TOPMed program and the NHGRI Genome Sequencing Program, WGS-based association analysis of complex traits remains a tremendous challenge due to the large number of rare variants, many of which are non-trait-associated neutral variants. External biological knowledge, such as functional annotations based on the ENCODE, Epigenomics Roadmap and GTEx projects, may be helpful in distinguishing causal rare variants from neutral ones; however, each functional annotation can only provide certain aspects of the biological functions. Our knowledge for selecting informative annotations *a priori* is limited, and incorporating non-informative annotations will introduce noise and lose power. We propose FunSPU, a versatile and adaptive test that incorporates multiple biological annotations and is adaptive at both the annotation and variant levels and thus maintains high power even in the presence of noninformative annotations. In addition to extensive simulations, we illustrate our proposed test using the TWINSUK cohort (n = 1,752) of UK10K WGS data based on six functional annotations: CADD, RegulomeDB, FunSeq, Funseq2, GERP++, and GenoSkyline. We identified genome-wide significant genetic loci on chromosome 19 near gene *TOMM40* and *APOC4-APOC2* associated with low-density lipoprotein (LDL), which are replicated in the UK10K ALSPAC cohort (n = 1,497). These replicated LDL-associated loci were missed by existing rare variant association tests that either ignore external biological information or rely on a single source of biological knowledge. We have implemented the proposed test in an R package “FunSPU”.

## Author summary

In recent years, large-scale whole-genome sequencing (WGS) data have been generated, such as those in the UK10K project and the ongoing NIH Trans-Omics for Precision Medicine (TOPMed) WGS program, providing unprecedented opportunities to investigate low-frequency variants and rare variants in association with complex diseases and traits. However, WGS-based association analysis of complex traits remains a tremendous challenge due to the large number of rare variants, many of which are non-trait-associated neutral variants. External biological knowledge, such as functional annotations based on the ENCODE, Epigenomics Roadmap and GTEx projects, can be helpful in distinguishing causal rare variants from neutral ones; however, each functional annotation can only provide certain aspects of the biological functions. To this end, we have proposed a versatile and adaptive association test, FunSPU, to exploit multiple sources of biological knowledge in the analysis of WGS data. We illustrate our proposed test using the TWINSUK cohort of UK10K WGS data based on six functional annotations. We identified genome-wide significant genetic loci associated with low-density lipoprotein, which are replicated in the UK10K ALSPAC cohort. These replicated loci were missed by existing rare variant association tests that either ignore external biological information or rely on a single source of biological knowledge.

## Introduction

In recent years, large-scale whole-exome sequencing and whole-genome sequencing (WGS) data have been generated, such as those in the Exome Sequencing Project [1], the UK10K project [2] and the ongoing NIH NHLBI Trans-Omics for Precision Medicine (TOPMed) WGS program [3], providing unprecedented opportunities to investigate low-frequency variants (minor allele frequency [MAF] between 1% and 5%) and rare variants (RVs; MAF < 1%) in association with complex diseases and traits. However, WGS-based association analysis of complex traits remains a tremendous challenge due to the large number of RVs, many of which are non-trait-associated neutral variants. External biological knowledge, such as functional annotations, might be informative to distinguish causal RVs from neutral ones. Some recent large-scale functional genomic studies, such as ENCODE [4], NIH Roadmap Epigenomics [5] and GTEx [6] projects, provide rich resources to use in characterizing the functional consequences of single nucleotide variants (SNVs), especially those in non-coding regions. Many approaches have been developed for functional annotations by integrating these data, e.g., CADD [7], GenoSkyline [8] and Eigen [9]; see Liu et al for a recent comparative review [10]. In WGS analysis, investigators may filter a subset of SNVs by annotations [2, 11], or use a single source of functional scores as weights in association tests to boost the statistical power [12–14]; however, each functional annotation can only provide a certain aspect of the biological functions, e.g., sequence conservation across species or biochemical activity of non-coding regions in a tissue. Our *a priori* knowledge to select the informative annotation(s) regarding a phenotype and genomic regions of interest is limited, and incorporating noninformative annotations will introduce noise and lose power.

To address this analytical challenge, we propose a family of versatile and powerful tests called “FunSPU” that allow for incorporating multiple functional annotations simultaneously in the adaptive sum of powered score (aSPU) test framework [15]. The fundamental idea of aSPU is to construct a general class of association tests, each of which is the most powerful under varying, yet unknown, local genetic architecture, then data-adaptively select the most significant test. Since each functional annotation system contains limited biological knowledge, multiple sources of functional annotations may provide complementary information. Therefore, a test that integrates multiple functional annotations simultaneously is potentially powerful. The proposed test is adaptive at both the annotation and variant levels and thus maintains high power even in the presence of noninformative annotations and a large number of neutral RVs. We also propose minimum *p*-value (minP) and Fisher’s meta-analysis-like approaches to combine the *p*-values with respect to multiple annotations. Moreover, to further increase the statistical power, we propose to incorporate a trait-specific global weight for each annotation based on partitioning the heritability.

Using extensive simulations and application to the UK10K WGS data [2], we compared our proposed FunSPU tests with the corresponding annotation-ignorant aSPU test as well as some existing RV association tests, such as the T5 burden test and SKAT [16]. We also compared our method with a recently published multiple functional annotation-based association test called functional score test (FST) [17]. Using the UK10K TWINSUK WGS cohort as the discovery sample (n = 1,752), we considered six functional annotations, CADD [7], RegulomeDB [18], FunSeq [19], Funseq2 [20], GERP++ [21] and GenoSkyline [8], and four quantitative traits, low-density lipoprotein (LDL), high-density lipoprotein (HDL), body mass index (BMI) and systolic blood pressure (SBP). We identified genome-wide significant genetic loci on chromosome 19 near gene *TOMM40* and *APOC4-APOC2* that are associated with LDL, which are replicated in the UK10K ALSPAC WGS cohort (n = 1,497). These replicated LDL-associated loci were missed by existing RV association tests that either ignore external biological information or rely on a single source of biological knowledge. We have implemented the proposed test in an R package “FunSPU”.

## Materials and methods

### Notations

Suppose that for subject *i* = 1, …, n, ***Y*** = (*Y*_*1*_, …, *Y*_*n*_) is the vector of a trait, and X_i_ = (*X*_*i1*_, …, *X*_*ik*_)’ is the vector of the genotype scores of *k* RVs, for example, from a gene or some genomic region. Here, we use additive coding for each RV; that is, X_*ij*_ is the count of the minor allele at RV *j* for subject *i*. For simplicity, we ignore other covariates in our model. We consider a generalized linear model (GLM):

$$g(E(Y_{i}))=\beta_{0}+\sum_{j=1}^{k}X_{ij}\beta_{j},$$

where g is a link function; for continuous *Y*_*i*_, g is the identity link *g*(*μ*) = *μ* and the GLM is reduced to a linear model, whereas g is the logit link $g(\mu )=log(\frac{\mu }{1-\mu })$ for binary *Y*_*i*_. For the purpose of exposition, we introduce our proposed tests in the linear model framework with a quantitative trait and no covariates, though the methods can be similarly extended to binary traits, and adjusted for covariates in the GLM and score function framework [15, 22, 23].

We test the null hypothesis *H*_0_: *β* = (*β*_*1*_, …, *β*_*k*_)’ = 0, that is, there is no association between any of the RVs and the trait under *H*_0_. Our proposed tests are based on the score vector *U* = (*U*_*1*_, …, *U*_*k*_)’ for *β* and its covariance matrix *V*,

$$U=\sum_{i=1}^{n}(Y_{i}-\bar{Y})X_{i},\;V=Cov(U|H_{0})=\bar{Y}(1-\bar{Y})\sum_{i=1}^{n}(X_{i}-\bar{X}){\left(X_{i}-\bar{X}\right)}^{\prime },$$

where $\bar{Y}$ and $\bar{X}$ are the sample means of the *Y*_i_’s and *X*_i_’s, respectively.

### Review of the data-adaptive aSPU test

Pan et al. [15] proposed a new adaptive test that retains high power across a wide range of varying, yet unknown, genetic architecture for the analysis of RVs. This test is based on a class of the SPU test:

$$T_{SPU(\gamma )}(U)=\sum_{j=1}^{k}U_{j}^{\gamma },$$

where γ≥1 is a positive integer. Suppose that we have a set of candidate values of γ in Γ, e.g., Γ = {1, 2, …, 8, ∞}, as used in our later experiments. It is known that SPU(1) is equivalent to the burden test, while SPU(2) is a variance-component score test equivalent to SKAT with a linear kernel. Importantly, as γ increases (as an even integer), the *SPU*(*γ*) test puts more weights on the larger component of *U* while gradually ignoring the remaining component. In particular, we have $T_{SPU(\gamma )}\propto {\left\|U\right\|}_{\gamma }={{(\sum_{j=1}^{k}\left|U_{j}\right|}^{\gamma })}^{\frac{1}{\gamma }}\to {\left\|U\right\|}_{\infty }=\max_{1\leq j\leq k}\left|U_{j}\right|$, as *γ*→∞. The SPU(∞) is closely related to the minP test (but ignores possibly varying variances of *U*_*j*_’s); the two tests often perform similarly [24]. Since the power of an *SPU*(*γ*) test depends on the choice of *γ* while the optimal choice of *γ* depends on the unknown true association pattern of the RVs to be tested, it would be desirable to data-adaptively choose the value of *γ*. To this end, the aSPU test takes the minimum *p*-value of the SPU(γ) tests as its test statistic: *T*_*aSPU*_ = min_*γ*∈Γ_*p*_*SPU*(*γ*_. In this case, *T*_*aSPU*_ is no longer a genuine *p*-value; we use resampling approaches such as residual permutation or parametric bootstrap to obtain its *p*-value.

### New test: FunSPU—A data-adaptive test incorporating multiple annotations

Our proposed test is in the data-adaptive aSPU test framework. Importantly, the proposed test is adaptive at both the annotation and SNV levels. Suppose that we have the score vector *U* = (*U*_*1*_, …, *U*_*k*_)’ for *k* RVs from a gene region or sliding window based on a linear regression model. Let 0≤*w*_*lj*_≤1 denote the functional score from the *l*th of *m* properly scaled annotations for the *j*th of *k* RVs. The proposed functional annotation-based SPU test is

$$T_{SPU-Fun(\gamma_{a},\gamma )}=\sum_{l=1}^{m}{\left[{\left(\sum_{j=1}^{k}{\left(w_{lj}\;U_{j}\right)}^{\gamma }\right)}^{\frac{1}{\gamma }}\right]}^{\gamma_{\alpha }},$$

where two positive integers γ≥1 and γ_*a*_≥1 respectively control the individual variants’ and annotations’ relative contributions to the overall test statistic; e.g., γ_*a*_ = 1 treats all annotations equally, while γ_*a*_ = ∞ only considers the most significant annotation. The inner sum of weighted *U*_*j*_ with power γ is the weighted SPU, and they are normalized to the power of 1/γ before being subjected to the outer sum with power γ_*a*_. Since the number of the RVs in this test statistic is identical across all *m* annotations, it is not necessary to further normalize the weighted SPU test by the number of RVs.

The intuition to use γ_*a*_ as the powers of the weighted SPU is similar to that for γ. In general, a smaller γ_*a*_, e.g., γ_*a*_ = 1, is more effective when there are more informative annotations, each of which is roughly equally discriminative regarding the deleteriousness of the RVs for the trait of interest. In contrast, a larger γ_*a*_ is preferred if there is only one or fewer informative annotations that can well distinguish causal variants from neutral ones for the trait. As γ_*a*_→∞, only the most significant weighted SPU is considered.

We aim to perform powerful tests when there are unknown association patterns of RVs and unknown informativeness of functional annotations. In practice, since we have no *a priori* knowledge about choosing γ and γ_*a*_, we need to conduct a grid search over a set of possible values of both γ and γ_*a*_. However, searching too many values will introduce extra variability and lead to reduced power. This effect was later confirmed when we used γ_*a*_∈{1,2,3,…,8,∞} and γ∈{1,2,3,…,8,∞} in some preliminary simulations. Based on the results of aSPU tests [15] and the feature of annotations, we decided to use γ_*a*_∈Γ_*a*_ = {1,2,4,8,∞} and γ∈Γ = {1,2,3,…,6} for the rest of the study. We retained γ_*a*_ = ∞ as an approximation to the minP test and ignored some higher values of γ since the results tend to be similar to γ = 6.

Given a set of γ and γ_*a*_, e.g., γ∈Γ = {1,2,3,…,6} and γ_*a*_∈Γ_*a*_ = {1,2,4,8,∞}, the proposed data-adaptive FunSPU test statistic is defined as

$$T_{FunSPU}=\underset{\gamma \in \Gamma ,\gamma_{a}\in \Gamma_{a}}{\min}p_{SPU-Fun(\gamma_{a},\gamma ),}$$

where $p_{SPU-Fun(\gamma_{a},\gamma )}$ is calculated by the resampling methods detailed below. Although the score vector U has an asymptotic normal distribution N(0, V), it is not easy to derive the asymptotic distribution of *T*_*FunSPU*_. Therefore, we propose using a single layer of permutations (without covariates) or residual permutations (with covariates) to obtain *p*-values as done in aSPU [15, 22]. Specifically, we first permute the original set of trait *Y* to obtain a new set of *Y*^*(b)*^ for *b* = 1, …, *B*. Then, we calculate the null score vector *U*^*(b)*^ and the corresponding test statistic $T_{SPU-Fun(\gamma_{a},\gamma )}^{\left(b\right)}=T_{SPU-Fun(\gamma_{a},\gamma )}(U^{\left(b\right)})$ as well as their *p*-values $p_{SPU-Fun(\gamma_{a},\gamma )}^{\left(b\right)}=\left[\sum_{b_{1}\neq b}I(\left|T_{SPU-Fun(\gamma_{a},\gamma )}^{\left(b_{1}\right)}|\geq |T_{SPU-Fun(\gamma_{a},\gamma )}^{\left(b\right)}\right|)+1\right]/B$. Therefore, we have $T_{FunSPU}^{\left(b\right)}=\min_{\gamma \in \Gamma ,\gamma_{a}\in \Gamma_{a}}p_{SPU-Fun(\gamma_{a},\gamma )}^{\left(b\right)}$, and the final *p*-value of the FunSPU test $p_{FunSPU}=\left[\sum_{b=1}^{B}I(T_{FunSPU}^{\left(b\right)}\leq T_{FunSPU})+1\right]/(B+1)$.

In the FunSPU test above, we ignored the possibly different variances of the score function component *U*_*j*_, for example, due to varying MAF of the RVs. On the other hand, previous research has shown that it may be beneficial to account for the heterogeneity of variances in the SPU framework [24]. Therefore, we further propose an inverse-variance weighted version of FunSPU:

$$T_{SPUw-Fun(\gamma_{a},\gamma )}=\sum_{l=1}^{m}{\left[{\left(\sum_{j=1}^{k}{\left(w_{lj}\;U_{j}/\sqrt{V_{jj}}\right)}^{\gamma }\right)}^{\frac{1}{\gamma }}\right]}^{\gamma_{\alpha }},$$

$$T_{FunSPUw}=\underset{\gamma \in \Gamma ,\gamma_{a}\in \Gamma_{a}}{\min}p_{SPUw-Fun(\gamma_{a},\gamma ),}$$

where *V*_*jj*_ is the *j*th diagonal element of *V* = Cov(*U*|*H*_0_) as given before.

### Alternative approaches to incorporating multiple functional annotations: aSPU_minP and aSPU_Fisher

We considered alternative approaches to incorporate multiple functional annotations into the aSPU test. In contrast to the two-level FunSPU approach, we can obtain modified aSPU tests via the score vector *U* weighted by each functional annotation, i.e., $T_{SPU(\gamma )}^{\left(l\right)}(U)=\sum_{j=1}^{k}{\left(w_{lj}\;U_{j}\right)}^{\gamma }\;and\;T_{aSPU}^{\left(l\right)}=\min_{\gamma \in \Gamma }p_{SPU(\gamma )}^{\left(l\right)}$, for *l* = 1, …, m. We can obtain the genuine *p*-value $p_{aSPU}^{\left(l\right)}$ by resampling methods. To combine multiple functional annotations, we can further employ some general approaches to combine multiple *p*-values, $p_{aSPU}^{\left(l\right)}$. For example, we can simply use $T_{aSPU\_minP}=\min_{1\leq l\leq m}p_{aSPU}^{\left(l\right)}$ as the test statistic of *m* modified aSPU tests. This aSPU_minP test is similar, but not exactly equivalent to the case of FunSPU with γ_*a*_ = ∞: the latter chooses the maximum $\left|T_{SPU(\gamma )}^{\left(l\right)}\right|$ and then uses resampling methods to obtain a genuine *p*-value directly, while the aSPU_minP test calculates the empirical *p*-value $p_{aSPU}^{\left(l\right)}$ first, and then uses the minimum *p*-value *T*_*aSPU*_*minP*_ as the new test statistic and resampling to calculate the final *p*-value.

Another common method for combining *p*-values is Fisher’s meta-analysis approach, i.e., $T_{aSPU\_Fisher}=-2\sum_{l=1}^{m}\ln\left(p_{aSPU}^{\left(l\right)}\right)$. If the *m p*-values were independent, *T*_*aSPU*_*Fisher*_ would follow a chi-squared distribution with 2*m* degrees of freedom. However, our $T_{aSPU}^{\left(l\right)}$ tests are correlated via the score vector *U*. Hence, we also use resampling approaches to calculate the final *p*-value. We can similarly apply the inverse-variance weighted method to aSPU_minP and aSPU_Fisher tests, respectively denoted as aSPUw_minP and aSPUw_Fisher.

Of note, aSPU_minP is closely related to the FST test [17]. Specifically when we restrict 𝛾 = 1,2, aSPU_minP is equivalent to FST_minP except for the up-weight of rarer variants and weighted sum approach to combine burden and dispersion test statistics in the latter, as compared to the minP approach in the former. Similarly, aSPU_Fisher is closely related to FST_Fisher.

### wtFunSPU: Extension of FunSPU to allow for global weighting of multiple annotations

In our proposed FunSPU test, we treated all *m* functional annotations equally *a priori* and completely relied on the data to adaptively combine multiple annotations in each test unit, for example, a sliding window. This may be less efficient in the presence of overall inferior or superior annotations for a trait of interest, in which case it would be desirable to globally down-weight inferior annotations (and up-weight superior annotations). To this end, we propose to modify the FunSPU test by introducing an annotation-level weight *ρ* = (*ρ*_1_,…,*ρ*_*m*_)′ and denote the modified test as wtFunSPU:

$$T_{wtSPU-Fun(\gamma_{a},\gamma )}^{*}=\sum_{l=1}^{m}{\left[\rho_{l}{\left(\sum_{j=1}^{k}{\left(w_{lj}\;U_{j}\right)}^{\gamma }\right)}^{\frac{1}{\gamma }}\right]}^{\gamma_{\alpha }},$$

$$T_{wtFunSPU}=\underset{\gamma \in \Gamma ,\gamma_{a}\in \Gamma_{a}}{\min}p_{wtSPU-Fun(\gamma_{a},\gamma ).}$$

Since we assume no *a priori* knowledge regarding the informativeness of a functional annotation for a given trait, we propose to estimate *ρ*_*l*_ based on some global correlation measure between the annotation weights, genotypes and phenotype. A promising approach is based on partitioning the heritability *h*^2^ by functional annotations [25]: a functional annotation is more informative for the trait of interest if SNVs with higher functional scores contribute to more heritability on average. Specifically, given an annotation, we first partition the genome-wide RVs based on Q discrete functional categories or percentiles of continuous functional scores; we then estimate the heritability $h_{q}^{2}$ for all SNVs in functional category *q = 1*,*…*, *Q*, using the GCTA tool [26]. We next compute the average per-SNV heritability $h_{q}^{2}/\#{SNV}^{\left(q\right)}$ for each annotation category *q* and regress $h_{q}^{2}/\#{SNV}_{q}$ on q to estimate the slope: $E(h_{q}^{2}/\#{SNV}^{\left(q\right)})=\beta_{0}+\beta q$, where *β* is used as the global weight *ρ* for the corresponding functional annotation in the wtFunSPU test. Prior to this calculation, we transform the functional annotation to positive integers *q = 1*,*…*, *Q* such that larger *q* corresponds to a more likely functional category. If a functional category has a very small number of SNVs or $h_{q}^{2}$ close to zero, this category is combined with a nearby category; see S1 to S4 Figs and S2 Table for details.

## Results

### Simulation setups

We conducted extensive simulations to evaluate and compare the performance of our proposed functional annotation-based tests with existing association tests for RVs. To make the simulation study representative of real RV data, we randomly selected 200 RVs from chr16:56.8M~57.1M of the UK10K TWINSUK genotype data of 1,718 unrelated individuals. MAFs of the selected RVs were no larger than 1%.

To evaluate power, we generated the simulated phenotypes as follows. First, we simulated 3 sets of informative annotations (*w*_1*j*_,*w*_2*j*_,*w*_3*j*_) and 3 sets of random annotations (*w*_4*j*_,*w*_5*j*_,*w*_6*j*_) independently (*j* = 1, 2, …, 200 ordered by genomic positions). We designated the first 100 RVs as causal variants (*j* = 1, 2, …, 100) and the remaining 100 RVs as neutral variants (*j* = 101, 102, …, 200). The informative annotations were generated from a uniform distribution *U*(0.4, 1) corresponding to causal variants and from *U*(0, 0.6) corresponding to neutral variants. All of the random annotations were generated from *U*(0, 1). Second, we randomly selected *k* = *k*_1_+ *k*_2_ RVs: *k*_1_ causal RVs from *j* = 1, 2, …, 100 and *k*_2_ neutral RVs from *j* = 101, 102, …, 200. Third, we used only informative annotations to calculate the effect size *β*_*j*_ = *c*_*β*_(*w*_1*j*_,*w*_2*j*_,*w*_3*j*_) for each causal RV. Fourth, the simulated phenotype was obtained from $Y_{i}=\sum_{j=1}^{k_{1}}{X_{ij}\beta }_{j}+\varepsilon_{i}$, where *ε*_*i*_ followed *N*(0,3) and *i* = 1,2,…,1718. Furthermore, to evaluate the globally weighted wtFunSPU test, we calculated the correlations between the sum of the genotypes weighted by each annotation and simulated phenotypes for each of the 1,000 simulation replications, and used the mean of the 1,000 correlations as the global weight of each annotation, i.e., $\rho_{l}=\bar{cor}(\tilde{Y},\sum_{j=1}^{k}w_{lj}{\tilde{X}}_{j})$ (*l* = 1, 2, …,6), where $\tilde{Y}$ and ${\tilde{X}}_{j}$ are the vectors of *Y*_*i*_ and *X*_*ij*_ in each replication correspondingly.

We considered two simulation scenarios. In scenario A, we used all three informative annotations, three random annotations and one dummy annotation (1’s for all RVs) in functional annotation-based tests (FunSPU, aSPU_minP, and others). To test the effect of more “noisy” annotations, we implemented scenario B, which used only one informative annotation, all three random annotations and one dummy annotation in the tests. In both scenarios A and B, we used identical procedure as above to generate simulated phenotypes *Y*_*i*_, and fixed *k*_1_ = 8 and *k*_2_ = {8, 16, 32, 64, 128}, respectively. We set *c*_*β*_ = 0.5 for tests that incorporated global weights and *c*_*β*_ = 1 for other tests.

To evaluate the type I error rate, we simulated *Y*_*i*_~*N*(0,3) (i = 1,2,…,1718), independent of *k* neutral RVs and 6 random annotations all from *U*(0,0.6) in each replication. We set increasing numbers of neutral RVs with *k =* {8, 16, 32, 64, 128}.

The empirical type I error rate was calculated based on 50,000 replications with the significance level α = 0.005, while the empirical power was calculated based on 1,000 replications for each scenario with α = 0.05. For permutation-based tests, 10,000 and 1,000 resamplings were conducted for each replication to evaluate type I error and power, respectively.

### Simulation results

As shown in Table 1, all the tests under comparison could control the type I error rate satisfactorily around 0.005, except for aSPU(w)_minP and aSPU(w)_Fisher tests, which were slightly inflated (between 0.006 and 0.007) with fewer number (e.g., 8) of neural variants. Besides Monte Carlo error, one possible reason for the slight inflation was that combining multiple annotations at the level of p-values might be sometimes numerically unstable in the presence of extreme p-values.

**Table 1:** Empirical type I error rates of various tests at significance level α = 0.005 for increasing number of neutral RVs with 50,000 simulation replications (B = 10,000 for resampling-based tests). Annotation-based tests were based on six random annotations. aSPU: adaptive sum of powered score test; aSPU_minP: combining multiple *p*-values of aSPU tests by minimum *p* approach; aSPU_Fisher: to combining multiple *p*-values of aSPU tests by Fisher’s meta-analysis approach; FunSPU: multiple functional annotation-based SPU test; wtFunSPU: global weighted FunSPU; T1: burden test of variants with MAF smaller than 1%; SKAT: the sequence kernel association test; (w): inverse-variance weighted score function in the SPU framework.

Test	No. of neutral RVs				
	8	16	32	64	128
aSPU	0.0059	0.0057	0.0050	0.0046	0.0043
aSPU_minP	0.0067	0.0058	0.0054	0.0062	0.0043
aSPUw_minP	0.0060	0.0053	0.0044	0.0056	0.0056
aSPU_Fisher	0.0061	0.0054	0.0059	0.0054	0.0047
aSPUw_Fisher	0.0062	0.0062	0.0051	0.0050	0.0047
FunSPU	0.0053	0.0047	0.0043	0.0045	0.0037
FunSPUw	0.0057	0.0062	0.0041	0.0050	0.0037
wtFunSPU	0.0045	0.0046	0.0046	0.0037	0.0042
wtFunSPUw	0.0047	0.0056	0.0039	0.0034	0.0050
T1	0.0052	0.0053	0.0049	0.0053	0.0055
SKAT	0.0051	0.0050	0.0053	0.0045	0.0038

**Fig 1:**
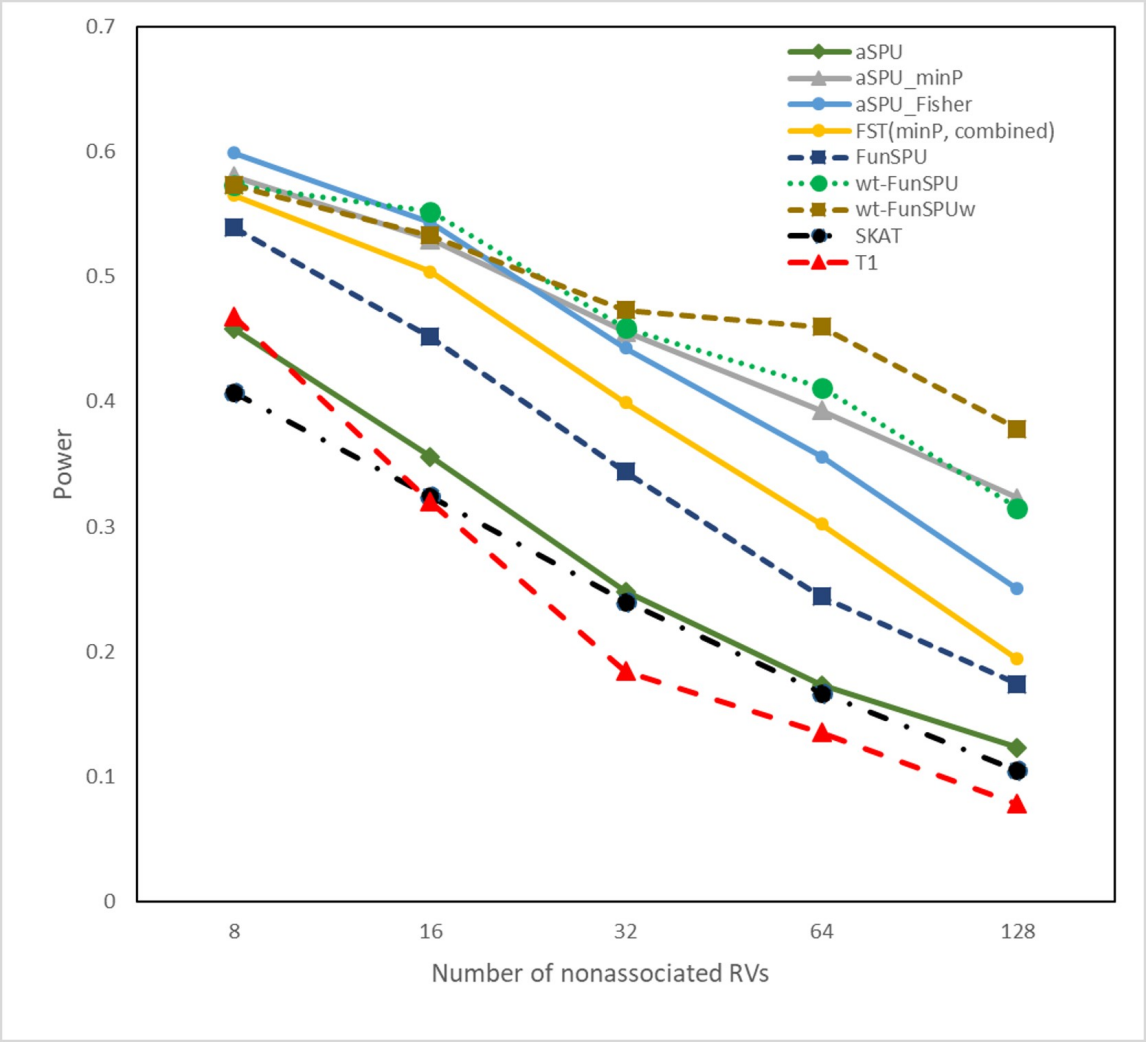
Empirical power of various tests for eight causal RVs and increasing number of nonassociated RVs at significance level α = 0.05. The incorporated annotations for association tests include all three informative annotations and three noninformative annotations (Scenario A). All the results were based on 1000 simulation replications.

Regarding power, we first considered scenario A (Fig 1), which was an advantageous scenario for our proposed tests since all three informative annotations together with three random annotations and one dummy annotation were used in the tests. The dummy annotation (constant 1) was supposed to retain the unweighted SPU in the adaptive tests, as in aSPU. Although the simulated annotations for causal and neutral RVs had modest differences, i.e., from *U*(0.4, 1) and *U*(0,0.6), respectively, the tests incorporating functional annotations, such as FunSPU, wtFunSPU, aSPU_minP and FST, always had higher power than tests that ignored functional annotations, such as aSPU, SKAT and T1. The FunSPU test appeared to be less powerful than aSPU_minP, suggesting a lack of efficiency in the former’s complete data-adaptive strategy to combine multiple annotations. On the other hand, wtFunSPU and wtFunSPUw outperformed aSPU_minP and FST, supporting the effectiveness of the global weighting scheme. Between the latter two, aSPU_minP had an increasing edge over FST in the presence of larger number of neural variants, due to its going beyond burden (SPU(*γ* = 1)) and variance-component (SPU(*γ* = 2)) tests with additional *γ* parameters. We also observed that the inverse-variance weighted tests always outperformed the original tests, e.g., wtFunSPUw versus wtFunSPU, and this advantage became more obvious with a higher proportion of neutral RVs. Lastly, the power of the aSPU_Fisher test was similar to that of the aSPU_minP test until the number of neural variants increased to 64 and 128, when the former became less powerful than the latter.

**Fig 2:**
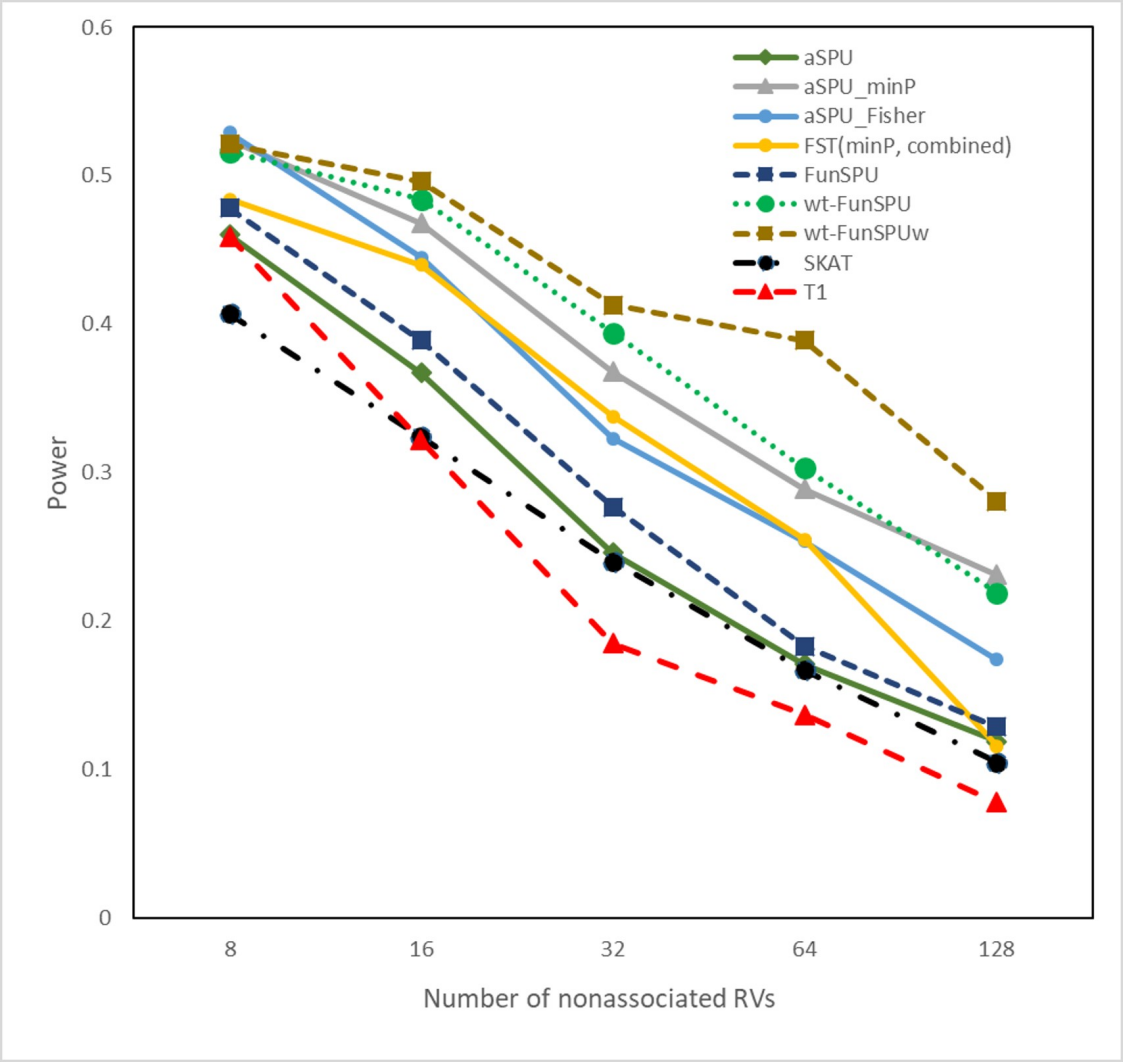
Empirical power of various tests for eight causal RVs and increasing number of nonassociated RVs at significance level α = 0.05. The incorporated annotations for association tests include one out of three informative annotations and three noninformative annotations (Scenario B). All the results were based on 1000 simulation replications.

Next, we considered a weaker scenario for our proposed tests. In scenario B (Fig 2), we used only one informative annotation, but all three random annotations and one dummy annotation in the tests. In this case, we had a higher proportion of “noisy” annotations in our tests. We observed that the FunSPU test was marginally more powerful than aSPU, SKAT and T1, but was less powerful than the aSPU_minP test by a large margin. In fact, scenario B was an advantageous scenario for the latter test, which only considered the most informative annotation. Similarly, aSPU_minP was more powerful than aSPU_Fisher when the number of neutral variants exceeded 16, due to the latter treating the one informative and three non-informative annotations equally. Finally, the globally weighted wtFunSPU and wtFunSPUw, especially the latter, were more powerful than the aSPU_minP and FST tests, again suggesting the benefit of globally down-weighting noninformative annotations.

We also compared the computational time needed for different methods. As shown in S1 Table, FunSPU and aSPU_minP were on par with aSPU, but were more computationally intensive than the asymptotic-based burden and SKAT tests. As shown in the real data analysis later on, by employing a step-up permutation strategy, we were able to perform genome-wide scan of WGS data with FunSPU and related tests.

### Application to the UK10K WGS data

To further evaluate the performance of our proposed tests on real data, we applied FunSPU and other state-of-the-art tests, including SKAT, T5 burden test and FST (combined test)[17], to association analysis of the UK10K WGS data with four complex quantitative traits: LDL, HDL, BMI and SBP. We used the TWINSUK samples as the discovery cohort and the ALSPAC samples as the replication cohort with n = 1706/1497 (TWINSUK/ ALSPAC), 1718/1497, 1752/1792 and 1740/1796, respectively, for LDL, HDL, BMI and SBP, after merging WGS genotype and phenotype data. After removing SNVs that did not pass quality control (QC) as done in the original UK10K analysis[2], as well as singletons and INDELs, we had a total of 10,979,027 RVs and low-frequency variants with MAF< 5% in the discovery cohort. Briefly, the UK10K WGS data QC included various low-level variant calling and filtering QC measures, variant-level QC to exclude variants with Hardy-Weinberg equilibrium (HWE) test p-value < 10^−6^, and sample-level QC to exclude samples in poor concordance with their corresponding GWAS data[2]. Since the discovery cohort TWINSUK only included women, we adjusted for age at baseline, but not gender, as a covariate in association testing in both discovery and replication cohorts.

We considered six types of functional annotations for RVs. CADD[7], FunSeq[19], FunSeq2[20], RegulomeDB[18] and GERP++[21] were extracted from the precomputed WGSA [27] library, and GenoSkyline (blood) annotation was generated from the region-based GenoSkyline library [8]. We re-scaled all annotations to numerical weights within the interval (0, 1), with larger weights corresponding to a greater likelihood of being functional (S5 Fig). Among the above annotations, rank scores for CADD, Funseq2, GenoSkyline and GERP++ were provided in the WGSA library [27], and the re-scaled score was defined as w = (raw rank score–min)/(max-min), where min and max were, respectively, the minimum and maximum raw rank scores for a given functional annotation. The RegulomeDB categories *s* = (1,2, …, 6) were transformed into (0, 1) by f(s) = (7-s)/6, whereas the Funseq categories *s* = (0,1,2, …, 6) were transformed by f(s) = (1+s)/7. We substituted the missing values or zero values with 0.01 (FunSeq, FunSeq2, RegulomeDB) or 0.0001 (GERP++). There was no missing value in CADD and GenoSkyline for the RVs considered here. S6 Fig shows the pairwise correlation coefficients among the 6 annotations: while some annotations were moderately correlated (r > 0.3), for example, GERP++ with CADD, and Funseq2 with RegulomeDB/Genoskyline, others were much less correlated. This suggests that multiple annotations may provide complementary information regarding the functional consequence of genetic variants, and it may be beneficial to incorporate them simultaneously in association analysis as proposed in the FunSPU framework here. Following the procedure proposed in Section 2.5, we calculated the phenotype-specific weight for each of the six annotations and used them as global weights in the wtFunSPU test. As shown in S1 to S4 Figs and S2 Table, RegulomeDB, Funseq and GenoSkyline tended to have consistently higher weights than GERP++, Funseq2 and CADD, while the numerical values and the relative magnitudes of the weights could vary across phenotypes.

We employed a sliding window approach to group RVs with a window length of 10k base pairs (bp) and a step size of 8.75k bp, resulting in 319,306 windows in total. Using the conservative Bonferroni procedure, we set the family-wise error rate at 0.05 with a significance level = 0.05/319306 = 1.56e-07, which equals 6.81 on the -log_10_ scale. To achieve this genome-wide significance level, we used a step-up permutation strategy [22, 28]. We first performed *B* = 10,000 permutations for all sliding windows and gradually increased *B*; if those sliding windows with estimated *p*-values <10/B, we increased *B* to 10 times the current value and re-estimated the *p*-values for these sliding windows. The number of permutations in the final stage was *B* = 10^8^. Of note, the variant-specific score functions in aSPU, aSPU_minP, FunSPU and wtFunSPU were not weighted by MAF, while those in aSPUw, aSPUw_minP, FunSPUw, and wtFunSPUw were inverse-variance weighted, where variants with lower MAF were up-weighted. By default, SKAT and FST used Beta(1,25) weights to up-weight variants with lower MAF [16, 17].

As shown in S7 to S10 Figs, the quantile-quantile plots for the proposed FunSPU tests were well behaved, with no discernible indication of global *p*-value inflation, suggesting that the FunSPU tests could control the type I error rate well in genome-wide scans. Table 2 shows all sliding windows with at least one genome-wide significant *p*-value in the TWINSUK discovery cohort by any of the association tests under consideration. To confirm our findings in the TWINSUK cohort, we performed replication analysis of the genome-wide significant sliding windows in the ALSPAC cohort. As shown in Table 2, four sliding windows were replicated for the corresponding phenotypes and association tests with a replication *p*-value < 0.05/24 = 2.1e-3 based on the Bonferroni correction for 24 sliding windows: 3 by at least one of the functional annotation-based tests (1 by wtFunSPU, 1 by FunSPU and aSPU_minP and 1 by aSPUw_minP) and one by the aSPU test. In contrast, none of the 6 sliding windows identified by the FST test in the discovery cohort was replicated; neither did SKAT nor T5 replicate any sliding window.

**Table 2:** Genome-wide significant sliding windows identified by various tests in the UK10K TWINSUK cohort and replication in the ALSPAC cohort of UK10K. Significant *p*-values are in boldface; only significant *p*-values in the ALSPAC cohort were reported (TWINSUK *p*-value/ALSPAC *p*-value shaded when both are significant). cMAF: cumulative minor allele frequency. Base pair (bp) position based on reference genome hg19.

Trait	Chr	Start position- Stop position (bp)	Gene(s)	cMAF	# SNVs	*p*-Values									
						Globally weighted		Not globally weighted		aSPU _minP	aSPUw _minP	aSPU	FST (combined)	SKAT	T5
						wtFun-SPU	wtFun -SPUw	Fun-SPU	Fun-SPUw						
HDL	1	39,070,016–39,080,016	Intergenic	0.14/0.33	39/38	4.3e-2	2.3e-3	3.3e-2	**<1e-8**	2.0e-2	3.2e-6	3.6e-2	1.2e-3	5.2e-2	2.0e-4
HDL	2	173,903,159–173,913,159	*RAPGEF4*	0.084/0.21	33/31	**<1e-8**	**<1e-8**	2.4e-6	**<1e-8**	4.9e-6	8.9e-6	2.4e-5	2.5e-6	1.1e-6	1.2e-4
HDL	3	63,804,000–63,814,000	*C3orf49*	0.02/0.17	37/36	2.9e-3	**<1e-8**	4.0e-2	1.8e-5	3.1e-2	4.5e-5	0.12	3.2e-3	1.6e-2	5.2e-2
HDL	5	6,548,648–6,558,648	Intergenic	0.001/0.32	59/59	0.13	**<1e-8**	0.18	**<1e-8**	6.2e-2**/ <1e-4**	<1e‐8/<1e‐4	0.15	2.0e-5	2.1e-3	0.19
HDL	5	6,557,398–6,567,398	Intergenic	0.34/0.26	48/43	4.3e-2	1.5e-2	0.13	**<1e-8**	9.5e-3	**<1e-8/**	0.24	**1.2e-8**	1.8e-2	0.81
LDL	3	102,287,400–102,297,400	Intergenic	0.25/0.19	56/39	4.4e-5	9.9e-3	**<1e-8**	3.6e-2	9.0e-6	1.3e-2	8.1e-6	1.3e-4	2.5e-5	4.5e-3
LDL	3	102,427,400–102,437,400	Intergenic	0.16/0.27	32/40	**<1e-8**	5e-8	**<1e-8**	1.3e-3	1.2e-6**/ <1e-4**	5.1e-5	2.5e-4	3.3e-7	1.3e-4	0.68
LDL	5	43,259,958–43,269,958	*NIM1K*	0.43/0.29	41/46	**9e-8**	**<1e-8**	6.5e-7	3.0e-7	1.7e-5**/ <1e-4**	8.2e-6	2.5e-5	1.5e-5	2.4e-3	4.4e-6
LDL	12	13,771,517–13,781,517	*GRIN2B*	0.18/0.23	43/48	3.5e-3	5.8e-4	0.80	3.5e-5	5.9e-6	1.7e-6	0.60	**2.4e-11**	0.23	0.96
LDL	12	13,780,267–13,790,267	*GRIN2B*	0.26/0.27	45/43	6.0e-2	8.0e-4	0.47	1.3e-4	1.0e-6	1.7e-6	0.33	**2.0e-11**	4.9e-2	0.53
LDL	19	45,387,096–45,397,096	*PVRL2/ TOMM40*	0.21/ 0.22	33/ 37	5.4e-2/ **1.8e-3**	0.10	3.5e-7/ **<1e-4**	5.0e-3/ **1.6e-3**	2.1e-7/ **<1e-4**	4.5e-3	5.0e‐8/<1e‐4	2.4e-4	2.4e-4/ **7.9e-6**	0.25
LDL	19	45,395,846–45,405,846	*TOMM40*	0.42/0.59	65/62	8.6e-3**/ <1e-4**	8.6e-2	3.0e‐8/<1e‐4	1.4e-4/ **1.9e-3**	<1e‐8/<1e‐4	5.8e-5**/ <1e-4**	5.0e-7	1.2e-4/ **1.0e-10**	4.7e-4/ **1.1e-6**	0.28
LDL	19	45,439,596–45,449,596	*APOC4-APOC2*	0.37/0.18	25/25	<1e‐8/<1e‐4	1.1e-4	1.2e-6**/ <1e-4**	6.9e-4	2.1e-5**/ <1e-4**	1.4e-4	2.3e-4	4.7e-5/ **2.4e-7**	1.1e-3/ **9.0e-4**	0.15
BMI	3	35,619,294–35,629,294	Intergenic	0.24/0.24	39/40	**<1e-8**	4.2e-5	5.0e-5	1.1e-4	3.9e-5	3.6e-5	8.0e-5	2.9e-6	4.4e-5	1.5e-5
BMI	4	22,825,237–22,835,237	Intergenic	0.18/0.24	47/48	1.3e-3	5.2e-5	1.8e-3	6.0e-5	1.6e-4	3.4e-4**/ <1e-4**	2.8e-3	**2.6e-8**	1.4e-6	3.0e-5
BMI	10	13,937,041–13,947,041	*FRMD4A*	0.28/0.30	49/53	8.5e-2	2.4e-3	0.16	1.4e-4	5.9e-4	2.4e-4	0.18	**1.9e-8**	0.12	7.2e-2
BMI	12	26,179,017–26,189,017	*RASSF8*	0.38/0.40	37/37	7.5e-2	1.2e-4	0.12	4.4e-4	2.1e-4	3.5e-4**/ <1e-4**	0.12	**7.5e-8**	6.6e-2	2.1e-4
BMI	15	42,935,528–42,945,528	*STARD9*	0.28/0.23	43/56	**<1e-8**	2.7e-6	**<1e-8**	1.2e-5	2.1e-6	4.5e-5	1.2e-6	4.8e-7	**8.4e-8**	**1.1e-7**
BMI	16	60,159,304–60,169,304	Intergenic	0.27/0.12	29/28	4.3e-4	**<1e-8**	2.5e-2	2.1e-4	6.1e-4**/ <1e-4**	6.4e-6**/ <1e-4**	5.1e-3	1.4e-5	0.15	3.0e-4
BMI	21	40,851,354–40,861,354	*SH3BGR*	0.26/0.17	32/26	4.4e-4	**<1e-8**	2.5e-3	1.1e-4	1.3e-3	6.7e-5 /**2.6e-3**	1.2e-3	3.9e-4	1.5e-2	3.4e-4
SBP	4	118,855,917–118,865,917	Intergenic	0.36/0.16	32/27	5.5e-2	**<1e-8**	0.68	8.1e-5	5.2e-2	4.2e-5**/ <1e-4**	0.75	2.5e-2	0.39	0.34
SBP	6	42,558,580–42,568,580	*UBR2*	0.96/0.095	28/22	**<1e-8**	1.3e-4	3.4e-4	5.6e-4	1.1e-6	1.6e-4	3.9e-4	1.7e-6	2.7e-3	7.8e-5
SBP	6	121,241,282–121,251,282	Intergenic	0.50/0.23	53/46	**<1e-8**	1.8e-4	1.2e-4	1.4e-3	1.6e-4	2.1e-3**/ <1e-4**	5.5e-4	3.0e-4	1.1e-4	7.0e-4
SBP	11	38,236,659–38,246,659	Intergenic	0.60/0.48	61/61	**<1e-8**	1.4e-4	8.6e-5	6.4e-6	2.1e-5	1.9e-4	3.8e-3	4.4e-6	8.5e-3	0.36

Significance threshold: *p*<1.56×10^−7^ for TWINSUK and *p*<2×10^−3^ for ALSPAC

Three of the four replicated sliding windows were close to each other on chromosome 19 around *TOMM40*, *APOE* and *APOC4-APOC2* genes. These loci have been previously identified and replicated to be associated with LDL by large-scale meta-analysis of GWAS common variants [29–31]. Numerous functional and genetic association studies have shown that *APOE* plays a central role in lipoprotein metabolism and neurodegeneration [32–34]. Specifically, *APOE* has three isoforms, 2, 3, and 4: *APOE2* is associated with elevated plasma LDL level and increased cardiovascular disease risk, whereas *APOE4* is associated with increased risk of Alzheimer’s disease[34]. While previous large-scale whole-exome sequencing and ExomeChip-based association studies did not identify exonic RVs in APOE associated with LDL[35, 36], a recent association analysis of 16,324 deep-coverage WGS samples from the TOPMed project identified LDL-associated rare non-coding variants upstream of *APOE*[37]. Here we were able to identify the *TOMM40/APOE* locus and additionally *APOC4-APOC2* locus that harbor LDL-associated RVs with fewer than a couple of thousand samples, suggesting that the power of the FunSPU test was boosted by incorporating external biological knowledge.

**Fig 3:**
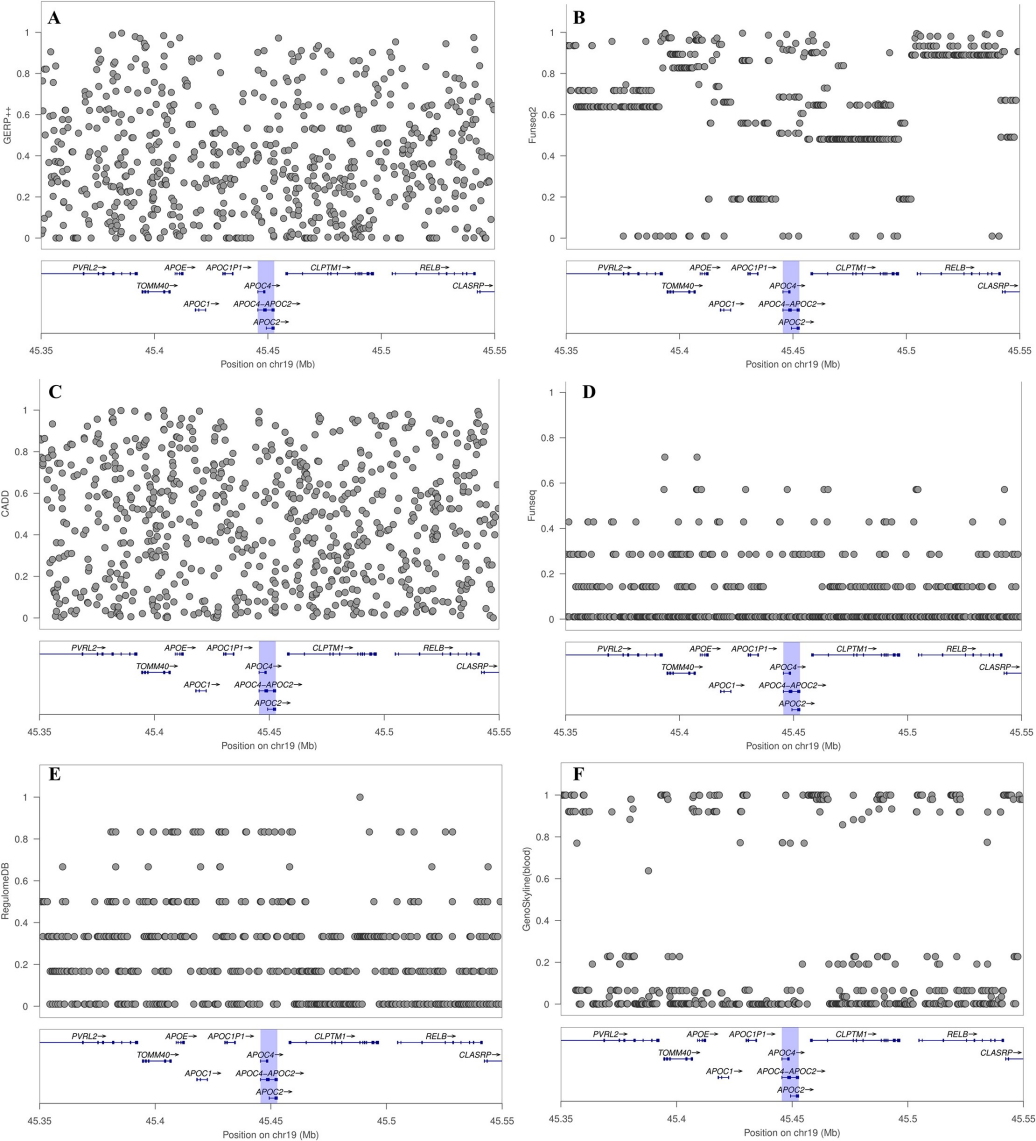
Rescaled scores of functional annotations: (A) GERP++, (B) Funseq2, (C) CADD, (D) Funseq, (E) RegulomeDB, and (F) GenoSkyline (blood) at the locus around gene *APOC4-APOC2*. The scores were rescaled to the interval [0, 1].

**Fig 4:**
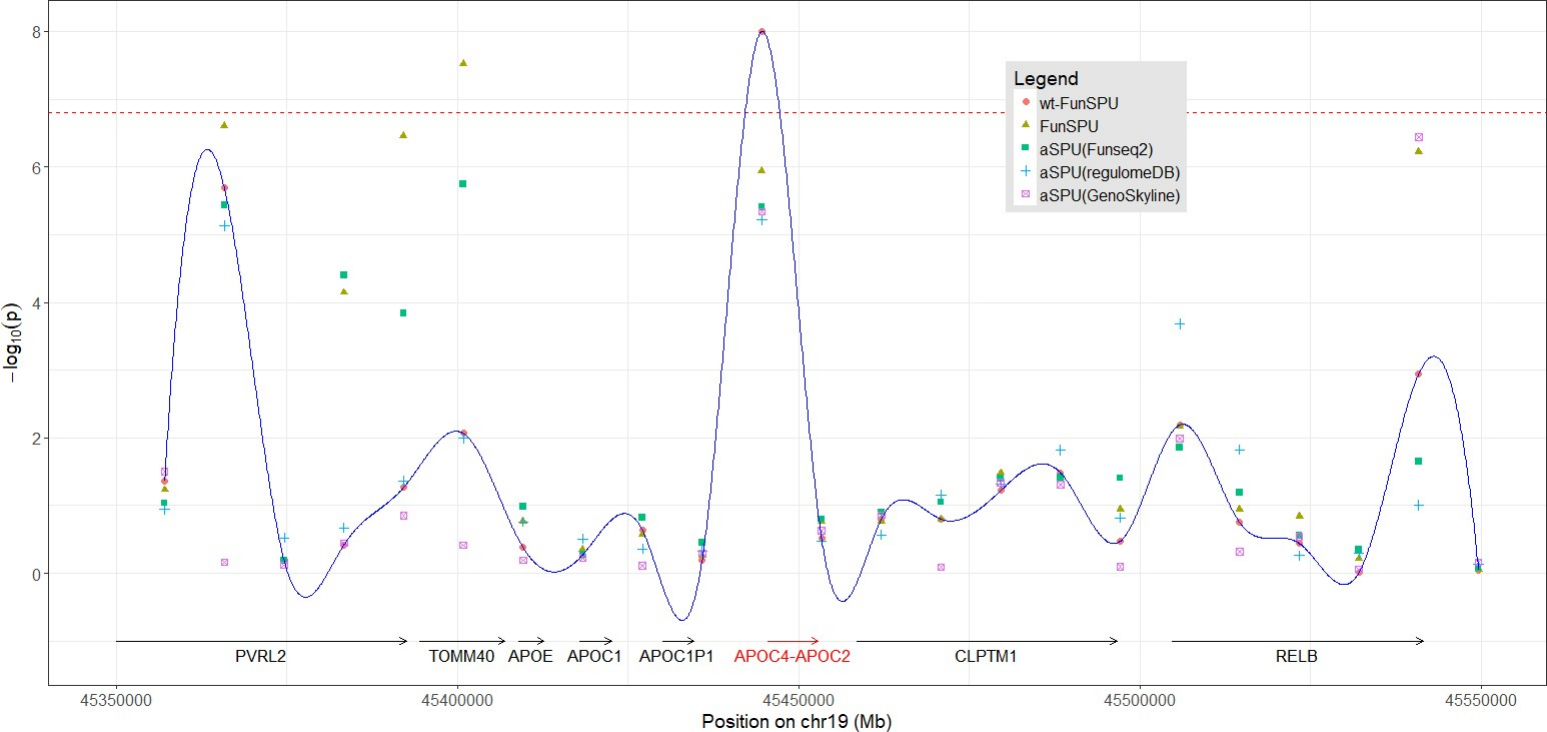
Association test results for LDL at the locus around gene *APOC4-APOC2*. The round points and the trace show the results from the globally weighted wtFunSPU test. Other points correspond to the results of FunSPU and single annotation-based aSPU (GERP++, Funseq2, RegulomeDB, GenoSkyline), respectively. Dashed line indicates the threshold of genome-wide significance level (*p* < 1.56e-7).

We also looked into the effects of multiple annotations on the FunSPU tests. Although some high scores were observed around the *TOMM40* and *APOC4-APOC2* gene regions for Funseq2, Funseq, RegulomeDB and GenoSkyline (Fig 3), they did not appear to be obviously different from those scores outside these two loci. Fig 4 shows the association signals of selected tests in this genomic region, whereas S11 Fig shows all individual annotation-based aSPU tests. As for *APOC4-APOC2*, three of the six annotations, namely, Funseq2, RegulomeDB and GenoSkyline (S11E, S11H and S11I Fig), positively contributed to the highly significant *p*-values of wtFunSPU and FunSPU (S11A and S11B Fig), although none of these individual annotation-based aSPU tests would reach the genome-wide significance threshold, demonstrating the benefit of integrating multiple functional annotations in the FunSPU framework. RegulomeDB and GenoSkyline also had higher global weights for LDL (S2B Table), which further boosted the *p*-value of the wtFunSPU test to the genome-wide significance level. As for *TOMM40*, Funseq2, CADD and GERP++ (S11E, S11F and S11D Fig) positively contributed to the genome-wide significance of FunSPU and aSPU_minP (S11A Fig and Table 2); whereas wtFunSPU missed this locus due to its low global weighting of these three annotations (S2B Table). This suggests that wtFunSPU, FunSPU and aSPU_minP may complement each other and may be used together in association analysis of WGS data.

To further investigate whether the *TOMM40* and *APOC4-APOC2* loci identified for LDL cholesterol were driven by coding RVs, we only retained nonsynonymous RVs in the original sliding windows in this region (13 nonsynonymous RVs out of total 784 RVs) and applied the aSPU test to each sliding window which had at least two RVs. For a sliding window with a single RV, we merged it with its neighboring sliding window. As shown in S12 Fig, none of the sliding windows had a p-value < 0.01, far less significant than the original association testing results (Fig 4). We therefore conclude that it is very unlikely the identified associations were driven by coding RVs.

## Discussion

We have proposed a versatile and adaptive association test, FunSPU, to exploit multiple sources of biological knowledge in the analysis of WGS data. It is adaptive at both the annotation and variant levels, and thus maintains high statistical power, even in the presence of noninformative annotations and a larger number of neutral variants. We have further proposed a globally weighted wtFunSPU test to more effectively down-weight less informative functional annotations in a trait-specific manner. Using the UK10K WGS data, we demonstrated that our proposed FunSPU test and its extensions, including the wtFunSPU and aSPU_minP tests, are more powerful tools to identify genome-wide significant loci than existing RV association tests that either ignore external biological information or rely on a single source of biological knowledge. The FunSPU family of tests would thus serve as a powerful and complementary tool for ongoing and future large-scale WGS studies, such as the NHLBI TOPMed project [3] of over 100,000 individuals and the UK Biobank [38] WGS project of 50,000 individuals. We have also summarized and compared the FunSPU family of tests in S3 Table.

The six functional annotations we considered here are diverse in terms of resources and features. For example, GERP++[21] is a sequence conservation score, whereas other annotations are ensemble scores based on integrating multiple sources of features, such as various functional genomic assays in the ENCODE project[4] and eQTL evidence. As demonstrated in S6 Fig, a majority of the annotations were only moderately correlated with each other, supporting our proposal to incorporate multiple annotations’ approximately orthogonal yet complementary information regarding the functional consequence of RVs in the framework of the FunSPU association test. The FunSPU test can easily incorporate additional functional annotations, including some newly developed ones [10], such as fathmm-MKL[39], Eigen/Eigen-PC[9] and DeepSEA[40].

To further de-noise noninformative annotations, we proposed a novel trait-specific measure based on partitioning the heritability and used it as a global weight for each annotation in the wtFunSPU test. Interestingly, our proposal is along the line of estimating group-specific weights in the context of weighted hypothesis testing [41, 42], though the latter is based on the mixture model, in contrast to the mixed model-based heritability partition here. Although it may look counterintuitive at first glance, our proposed data-dependent global weights actually did not inflate the type I error rates in both simulations (Table 1) and the real data analysis, as evidenced by the QQ plots of wtFunSPU (S7 to S10 Figs). The reason is that we used a much larger number of observations, i.e., RVs across the whole genome, to estimate a few annotation category-specific heritability parameters h^2^, based on which we derived a single global weight. This is in line with the “sieve principle”, which justifies using aggregated data to estimate a much smaller number of weights and then using them in subsequent hypothesis testing of small units of data (e.g., genes or sliding windows) with controlled family-wise error rate [42, 43]. Our proposed measure also has the potential to be used to compare the discriminative performance of whole-genome annotations for a complex trait of interest, for which known deleterious and neutral variants are rarely available (see S2 Table). This warrants further investigation. We have also applied the LD score regression method [44] to calculate the weights for common variants (MAF>5%) using the UK10K TWINSUK WGS data. As shown in S4 Table, the weights were largely qualitatively similar to those derived from RVs, suggesting that our proposed strategy to infer the global weighting of annotations is quite robust.

We have some practical considerations for our proposed tests. First, some functional annotations are not well-defined across the whole genome, resulting in relatively high missing data rates, for example, 68% for Funseq; the missing scores may reduce the reliability of annotation-based association tests. On the other hand, considering multiple complementary functional annotations simultaneously may at least partially remedy the problem of missing information. Second, by employing parallel computing and a step-up residual permutation strategy for the FunSPU family of tests, we are able to perform computationally feasible genome-wide scans for WGS data. For example, in the UK10K TWINSUK WGS data application, it took 24 hours for 500 computing cores to complete the sliding window-based FunSPU scan in R, including 10^8^ residual permutations for the top sliding windows to reach the genome-wide significance threshold. We expect that further implementation of the core functions in the C language should reduce the computational burden to a more affordable level. In addition, it would be desirable to develop some asymptotic theory and test to save the computational time. Of note, some asymptotic theory has been developed for the aSPU test in the context of testing two high-dimensional means for common variants [45]; however, extension to rare variants and the FunSPU test proposed here is not trivial and warrants future research.

We have implemented the proposed FunSPU test and its extensions in an R package “FunSPU”, available at https://github.com/sputnik1985/FunSPU, and to be posted to R/CRAN.

## Supporting information

S1 Fig Heritability per SNV (h^2^/#SNV) of HDL sorted by category of functional annotation score (TWINSUK cohort). (PDF)

S2 Fig Heritability per SNV (h^2^/#SNV) of LDL sorted by category of functional annotation score (TWINSUK cohort). (PDF)

S3 Fig Heritability per SNV (h^2^/#SNV) of BMI sorted by category of functional annotation score (TWINSUK cohort). (PDF)

S4 Fig Heritability per SNV (h^2^/#SNV) of SBP sorted by category of functional annotation score (TWINSUK cohort). (PDF)

S5 Fig Distributions of genome-wide functional scores for rare variants (MAF < 5%) in the UK10K TWINSUK cohort. (PDF)

S6 Fig Pairwise correlation of rescaled scores of functional annotations across the whole genome. (PDF)

S7 Fig Global quantile-quantile (QQ) plots for association analysis of rare variants with HDL in the UK10K TWINSUK cohort: (A) FunSPU (genomic control λ = 1.004), (B) FunSPUw (λ = 1.076), (C) wtFunSPU with global weights (λ = 1.004), and (D) wtFunSPUw with global weights (λ = 1.047).
(PDF)

S8 Fig Global QQ plots for association analysis of rare variants with LDL in the UK10K TWINSUK cohort: (A) FunSPU (genomic control λ = 0.611), (B) FunSPUw (λ = 0.805), (C) wtFunSPU with global weights (λ = 0.642), and (D) wtFunSPUw with global weights (λ = 0.789).
(PDF)

S9 Fig Global QQ plots for association analysis of rare variants with BMI in the UK10K TWINSUK cohort: (A) FunSPU (genomic control λ = 0.604), (B) FunSPUw (λ = 0.825), (C) wtFunSPU with global weights (λ = 0.635), and (D) wtFunSPUw with global weights (λ = 0.825).
(PDF)

S10 Fig Global QQ plots for association analysis of rare variants with SBP in the UK10K TWINSUK cohort: (A) FunSPU (genomic control λ = 0.601), (B) FunSPUw (λ = 0.821), (C) wtFunSPU with global weights (λ = 0.631), and (D) wt-FunSPUw with global weights (λ = 0.793).
(PDF)

S11 Fig LocusZoom plots of association test results for LDL at the locus around TOMM40 and APOC4-APOC2 in the UK10K TWINSUK cohort: (A) FunSPU, (B) wtFunSPU incorporating global weights, (C) aSPU, and (D)-(I) aSPU incorporating a single functional annotation: (D) GERP++, (E) Funseq2, (F) CADD, (G) Funseq, (H) RegulomeDB, and (I) GenoSkyline (blood).
(PDF)

S12 Fig Association test results for coding rare variants (MAF < 5%) in association with LDL around genes *TOMM40*, *APOE*, and *APOC4-APOC2*. (PDF)

S1 Table Computational time needed for selected methods under comparison. (PDF)

S2 Table List of heritability of RVs by each category of functional annotation (TWINSUK cohort). The corresponding phenotypes: (A) HDL, (B) LDL, (C) BMI, and (D) SBP.
(PDF)

S3 Table Summary and comparison of the proposed tests. (PDF)

S4 Table List of heritability of common variants (CVs; MAF>5%) by each category of functional annotation (TWINSUK WGS cohort) estimated by LD score regression. (PDF)
