# Supplementary material for: FunSPU: A versatile and adaptive multiple functional annotation-based association test of whole-genome sequencing data
Source: PLoS Genet. 2019 Apr 29;15(4):e1008081. doi: 10.1371/journal.pgen.1008081 (PMC6508749; doi:10.1371/journal.pgen.1008081)

Supplemental Figure S4. Heritability per SNV (h^2/#SNV) of SBP sorted by category of functional annotation score (TWINSUK cohort). Dashed lines represent linear regression results (categories merged).

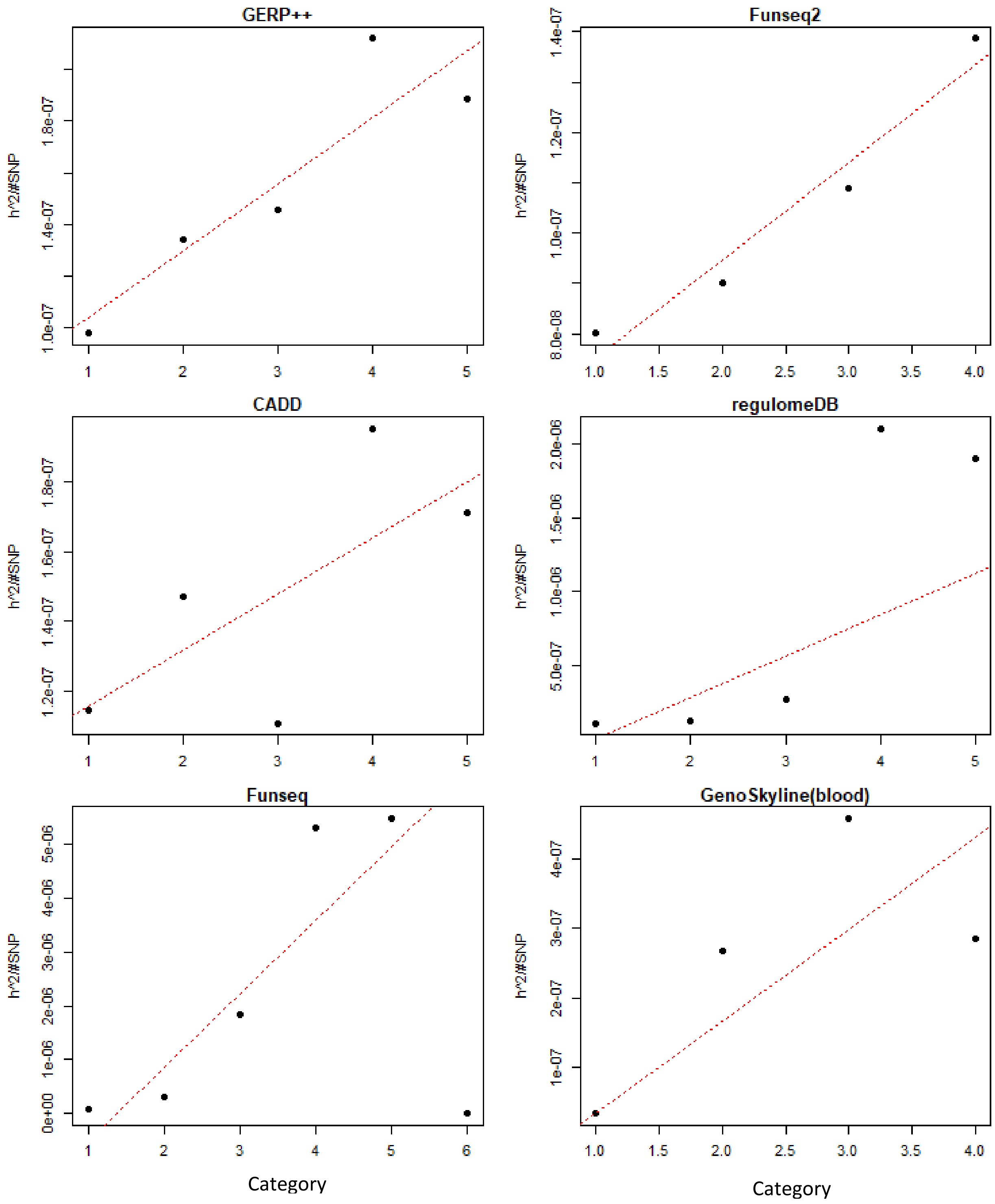

Supplement: S4 Fig — (PDF) [file pgen.1008081.s004.pdf]
