# Supplementary material for: FunSPU: A versatile and adaptive multiple functional annotation-based association test of whole-genome sequencing data
Source: PLoS Genet. 2019 Apr 29;15(4):e1008081. doi: 10.1371/journal.pgen.1008081 (PMC6508749; doi:10.1371/journal.pgen.1008081)

Supplemental Figure S6. Pairwise correlation of rescaled scores of functional annotations across the whole genome. The correlation plot was generated by R package corrplot.

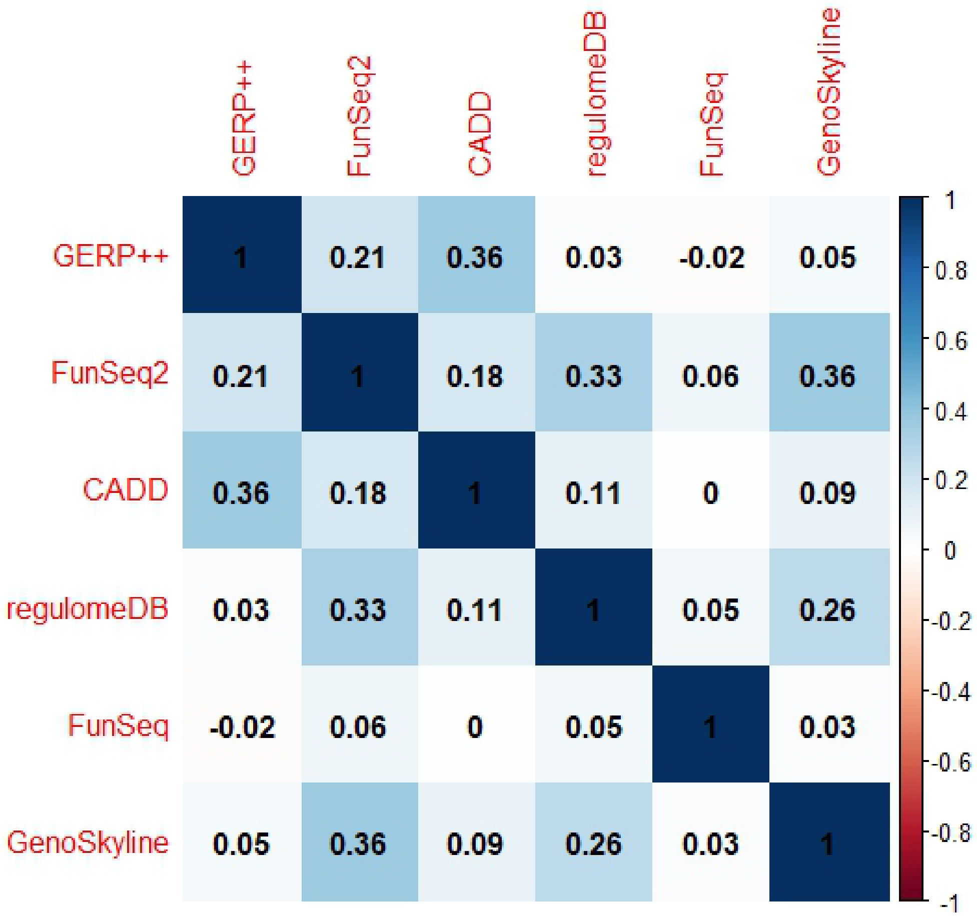

Supplement: S6 Fig — (PDF) [file pgen.1008081.s006.pdf]
