# Supplementary material for: FunSPU: A versatile and adaptive multiple functional annotation-based association test of whole-genome sequencing data
Source: PLoS Genet. 2019 Apr 29;15(4):e1008081. doi: 10.1371/journal.pgen.1008081 (PMC6508749; doi:10.1371/journal.pgen.1008081)

Supplemental Figure S7. Global quantile-quantile (QQ) plots for association analysis of rare variants with HDL in the UK10K TWINSUK cohort: (a) FunSPU (genomic control λ = 1.004), (b) FunSPUw (λ = 1.076), (c) wtFunSPU with global weights (λ = 1.004), and (d) wtFunSPUw with global weights (λ = 1.047).

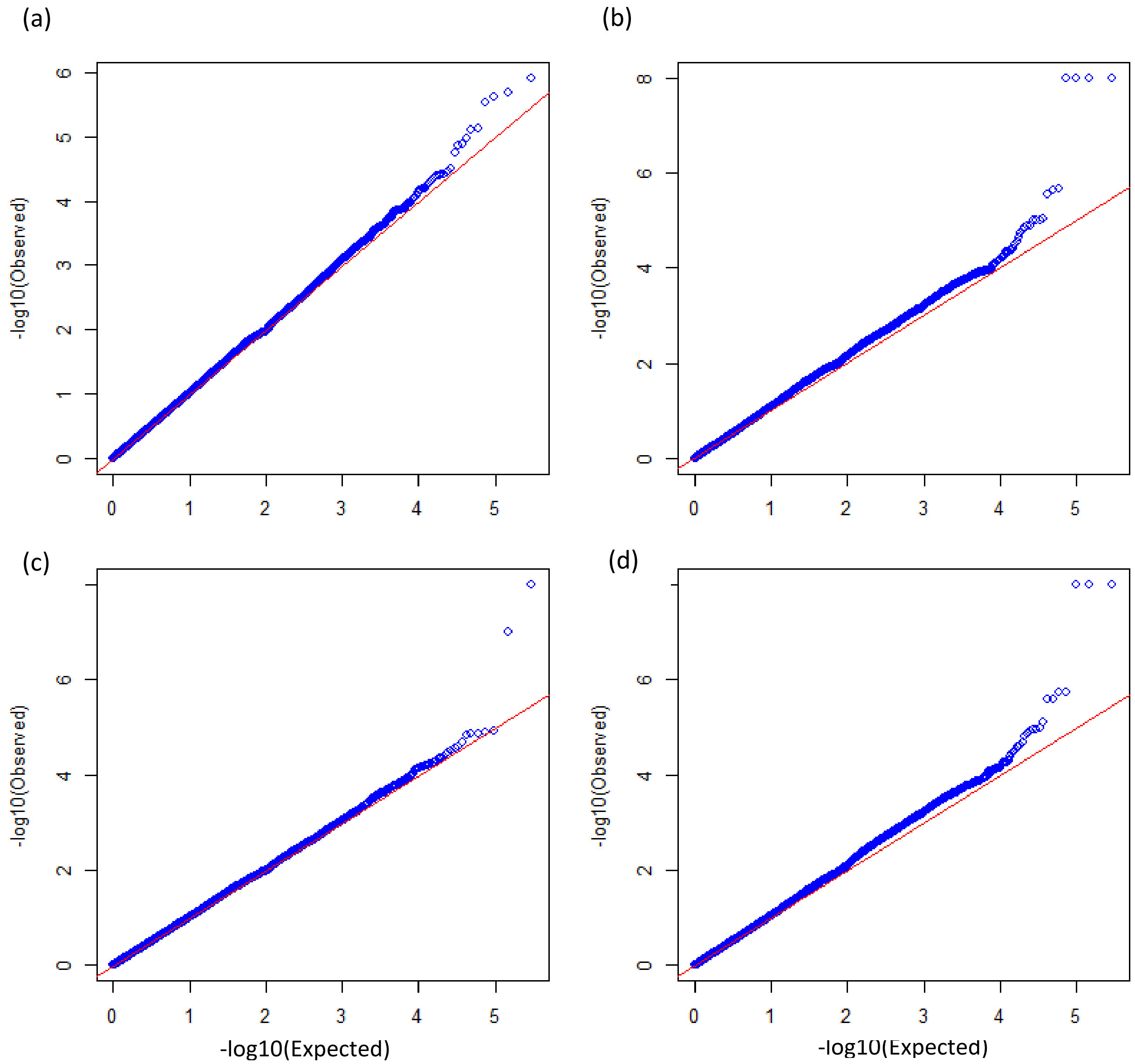

Supplement: S7 Fig — Global quantile-quantile (QQ) plots for association analysis of rare variants with HDL in the UK10K TWINSUK cohort: (A) FunSPU (genomic control λ = 1.004), (B) FunSPUw (λ = 1.076), (C) wtFunSPU with global weights (λ = 1.004), and (D) wtFunSPUw with global weights (λ = 1.047). (PDF) [file pgen.1008081.s007.pdf]
