# Supplementary material for: FunSPU: A versatile and adaptive multiple functional annotation-based association test of whole-genome sequencing data
Source: PLoS Genet. 2019 Apr 29;15(4):e1008081. doi: 10.1371/journal.pgen.1008081 (PMC6508749; doi:10.1371/journal.pgen.1008081)

Supplemental Figure S9. Global QQ plots for association analysis of rare variants with BMI in the UK10K TWINSUK cohort: (a) FunSPU (genomic control λ = 0.604), (b) FunSPUw (λ = 0.825), (c) wtFunSPU with global weights (λ = 0.635), and (d) wtFunSPUw with global weights (λ = 0.825).

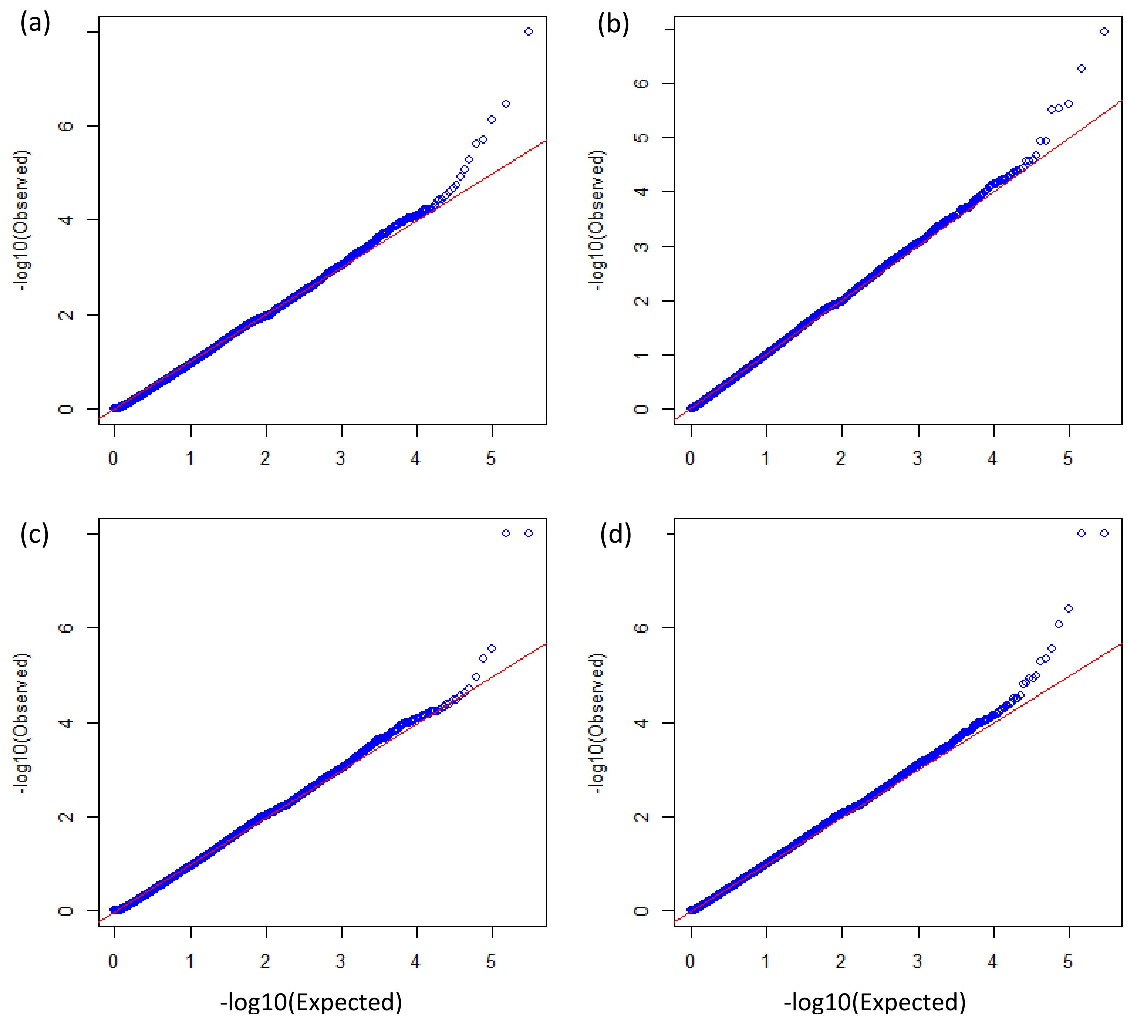

Supplement: S9 Fig — Global QQ plots for association analysis of rare variants with BMI in the UK10K TWINSUK cohort: (A) FunSPU (genomic control λ = 0.604), (B) FunSPUw (λ = 0.825), (C) wtFunSPU with global weights (λ = 0.635), and (D) wtFunSPUw with global weights (λ = 0.825). (PDF) [file pgen.1008081.s009.pdf]
