# Supplementary material for: FunSPU: A versatile and adaptive multiple functional annotation-based association test of whole-genome sequencing data
Source: PLoS Genet. 2019 Apr 29;15(4):e1008081. doi: 10.1371/journal.pgen.1008081 (PMC6508749; doi:10.1371/journal.pgen.1008081)

Supplemental Figure S10. Global QQ plots for association analysis of rare variants with SBP in the UK10K TWINSUK cohort: (a) FunSPU (genomic control λ = 0.601), (b) FunSPUw (λ = 0.821), (c) wtFunSPU with global weights (λ = 0.631), and (d) wt-FunSPUw with global weights (λ = 0.793).

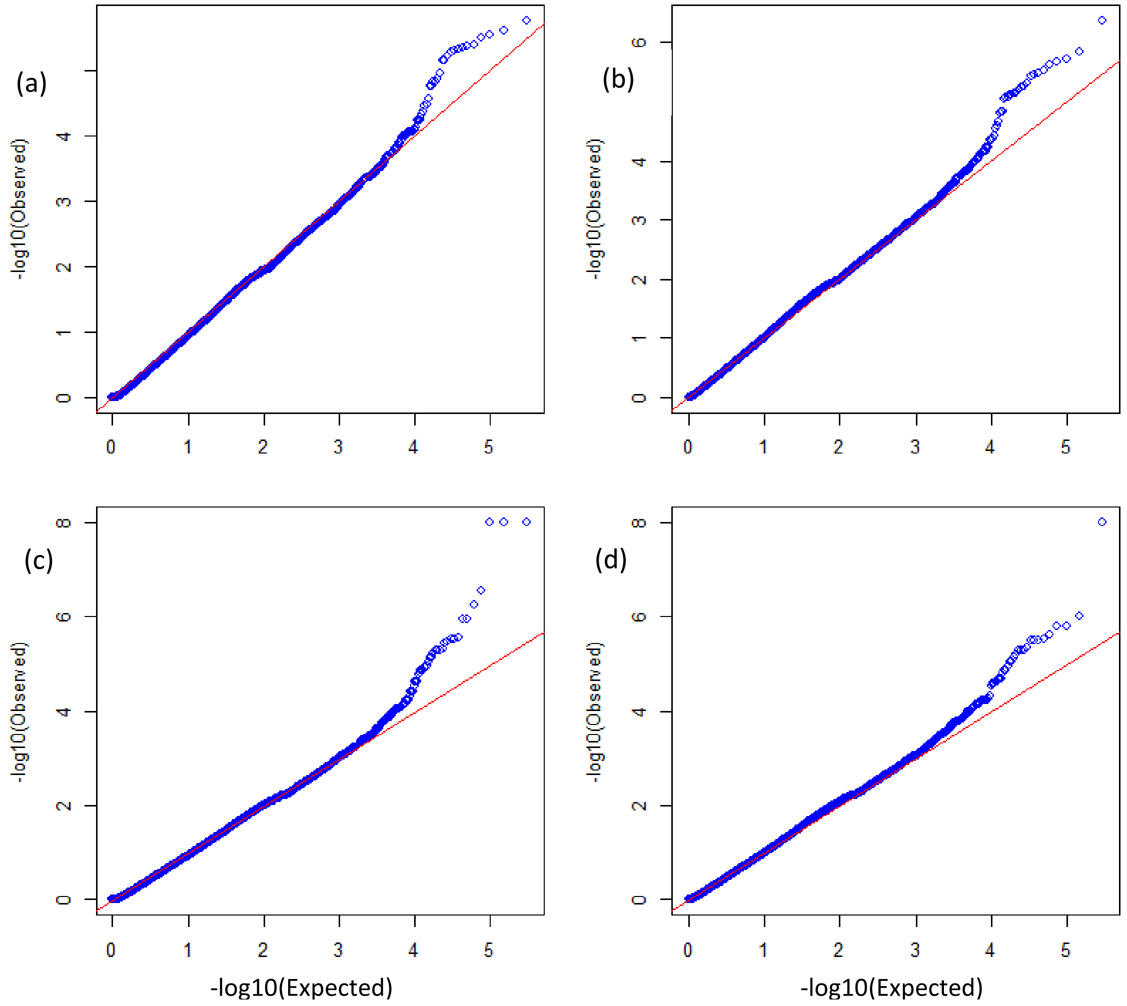

Supplement: S10 Fig — Global QQ plots for association analysis of rare variants with SBP in the UK10K TWINSUK cohort: (A) FunSPU (genomic control λ = 0.601), (B) FunSPUw (λ = 0.821), (C) wtFunSPU with global weights (λ = 0.631), and (D) wt-FunSPUw with global weights (λ = 0.793). (PDF) [file pgen.1008081.s010.pdf]
