# Supplementary material for: FunSPU: A versatile and adaptive multiple functional annotation-based association test of whole-genome sequencing data
Source: PLoS Genet. 2019 Apr 29;15(4):e1008081. doi: 10.1371/journal.pgen.1008081 (PMC6508749; doi:10.1371/journal.pgen.1008081)

Supplemental Figure S11. LocusZoom plots of association test results for LDL at the locus around *TOMM40* and *APOC4-APOC2* in the UK10K TWINSUK cohort: (A) FunSPU, (B) wtFunSPU incorporating global weights, (C) aSPU, (D)-(I) aSPU incorporating a single functional annotation: (D) GERP++, (E) Funseq2, (F) CADD, (G) Funseq, (H) RegulomeDB, and (I) GenoSkyline (blood).

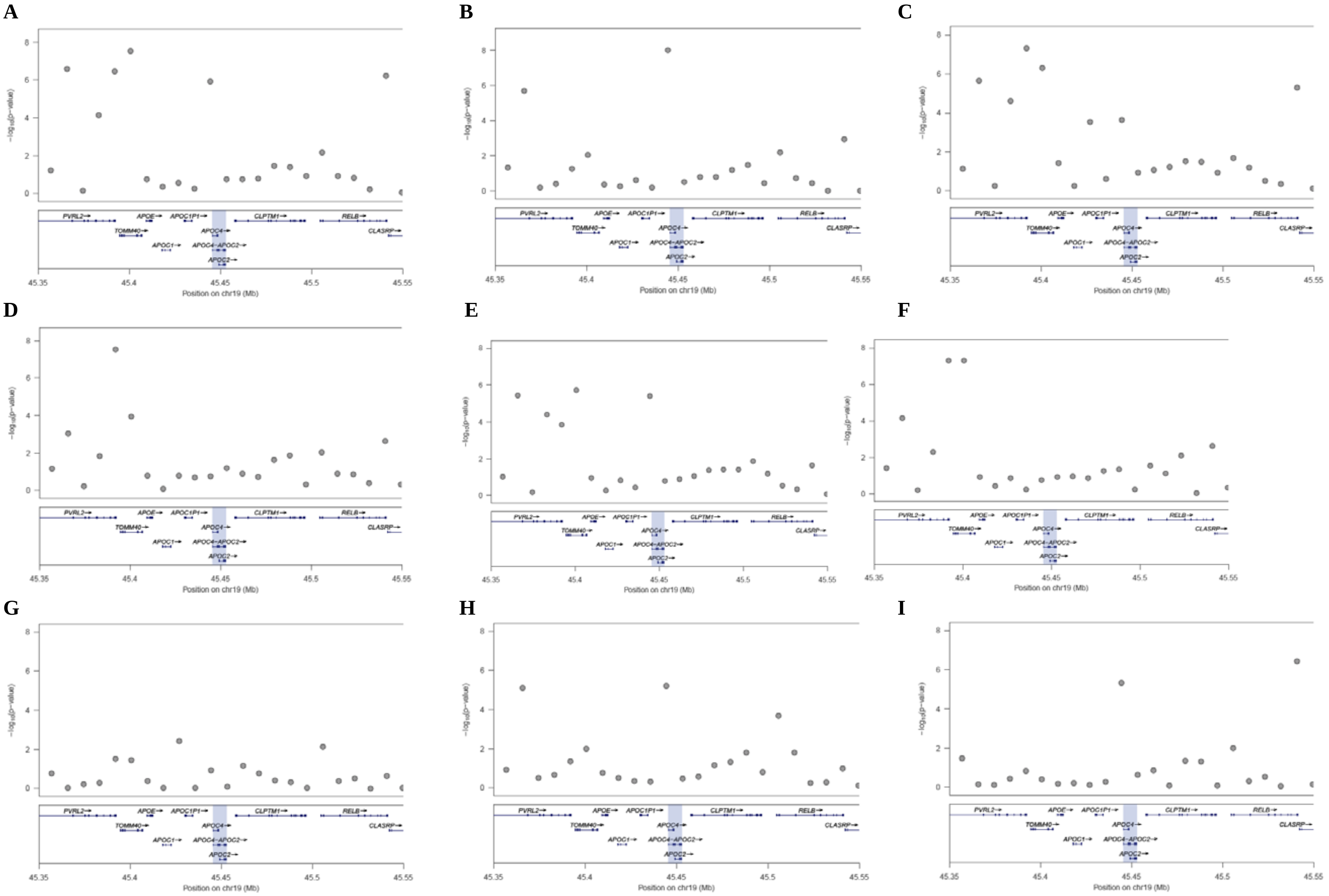

Supplement: S11 Fig — LocusZoom plots of association test results for LDL at the locus around TOMM40 and APOC4-APOC2 in the UK10K TWINSUK cohort: (A) FunSPU, (B) wtFunSPU incorporating global weights, (C) aSPU, and (D)-(I) aSPU incorporating a single functional annotation: (D) GERP++, (E) Funseq2, (F) CADD, (G) Funseq, (H) RegulomeDB, and (I) GenoSkyline (blood). (PDF) [file pgen.1008081.s011.pdf]
