# Supplementary material for: FunSPU: A versatile and adaptive multiple functional annotation-based association test of whole-genome sequencing data
Source: PLoS Genet. 2019 Apr 29;15(4):e1008081. doi: 10.1371/journal.pgen.1008081 (PMC6508749; doi:10.1371/journal.pgen.1008081)

Supplemental Figure S12. Association test results for coding rare variants (MAF < 5%) in association with LDL around genes *TOMM40*, *APOE*, and *APOC4-APOC2*. Red dots correspond to –log10(aSPU p-value), and blank dots mark the locations of nonsynonymous rare variants. Dashed line indicates the threshold of genome-wide significance level (p < 1.56e-7).

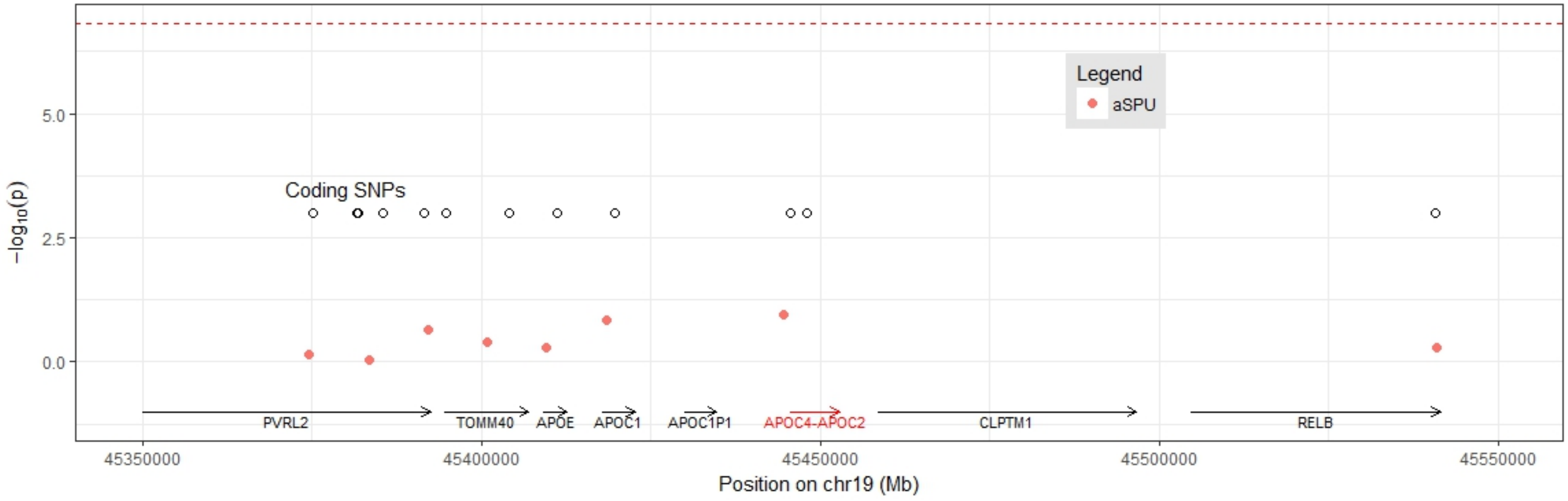

Supplement: S12 Fig — (PDF) [file pgen.1008081.s012.pdf]
