# Supplementary material for: FunSPU: A versatile and adaptive multiple functional annotation-based association test of whole-genome sequencing data
Source: PLoS Genet. 2019 Apr 29;15(4):e1008081. doi: 10.1371/journal.pgen.1008081 (PMC6508749; doi:10.1371/journal.pgen.1008081)
Supplement: S1 Table — (PDF) [file pgen.1008081.s013.pdf]

Supplemental Table S1. Computational time needed (mean and standard deviation (SD) with 32 rare variants (RVs) in an RV-set) for selected methods under comparison in the simulation study of power (Figure 1; Scenario A for power evaluation).

Computation time (in seconds)	Mean	SD
aSPU	0.268	0.054
aSPU_minP	1.923	0.118
FunSPU	0.804	0.070
SKAT	0.033	0.022
Burden (T1)	0.024	0.015
