# Supplementary material for: FunSPU: A versatile and adaptive multiple functional annotation-based association test of whole-genome sequencing data
Source: PLoS Genet. 2019 Apr 29;15(4):e1008081. doi: 10.1371/journal.pgen.1008081 (PMC6508749; doi:10.1371/journal.pgen.1008081)
Supplement: S2 Table — The corresponding phenotypes: (A) HDL, (B) LDL, (C) BMI, and (D) SBP. (PDF) [file pgen.1008081.s014.pdf]

Supplemental Table S2. List of heritability of RVs by each category of functional annotation (TWINSUK cohort). The *italicized* categories of each annotation were merged as one category. The corresponding phenotypes: (A) HDL, (B) LDL, (C) BMI, and (D) SBP

(A) High-density lipoprotein (HDL)

Annotation	value	h^2 (RV)	#SNV	h^2/#SNV	Normalized (by GERP++=1)	Regression Slopes
GERP++	1	0.391	2610225	1.497E-07	1.000	0.204
	2	0.454	2432456	1.868E-07	1.247	
	3	0.399	1878023	2.125E-07	1.419	
	4	0.401	1781008	2.253E-07	1.504	
	5	0.455	1603753	2.834E-07	1.893	
Funseq2	1	0.346	4208678	8.224E-08	0.549	0.177
	2	0.484	2483215	1.949E-07	1.302	
	3	0.396	2311991	1.714E-07	1.145	
	4	0.332	1861326	1.784E-07	1.191	
CADD	1	0.410	2563253	1.600E-07	1.068	0.238
	2	0.427	2446753	1.744E-07	1.165	
	3	0.416	2310764	1.801E-07	1.203	
	4	0.420	1905380	2.204E-07	1.472	
	5	0.552	1752877	3.149E-07	2.103	
RegulomeDB	1	0.438	3083174	1.420E-07	0.948	2.502
	2	0.495	2111757	2.345E-07	1.566	
	3	*0.417*	*556547*	*7.497E-07*	*5.006*	
	4	*0.423*	*136034*	*3.110E-06*	*20.770*	
	5	*0.224*	*187380*	*1.196E-06*	*7.988*	
Funseq	1	0.487	4990794	9.759E-08	0.652	15.170
	2	0.470	1080633	4.347E-07	2.903	
	3	0.412	228382	1.805E-06	12.054	
	4	*0.313*	*29950*	*1.044E-05*	*69.685*	
	5	*0.000*	*5027*	*1.989E-10*	*0.001*	
	6	*0.000*	*280*	*3.571E-09*	*0.024*	
GenoSkyline (blood)	1	0.459	9702179	4.726E-08	0.316	5.359
	2	*0.212*	*245856*	*8.643E-07*	*5.772*	
	3	*0.147*	*120694*	*1.222E-06*	*8.159*	
	4	*0.348*	*910298*	*3.820E-07*	*2.551*	

(B) Low-density lipoprotein (LDL)

Annotation	value	h^2 (RV)	#SNV	h^2/#SNV	Normalized (by GERP++=1)	Regression Slopes
GERP++	1	0.349	2610225	1.339E-07	1.000	0.046
	2	0.406	2432456	1.669E-07	1.246	
	3	0.371	1878023	1.974E-07	1.474	
	4	0.275	1781008	1.545E-07	1.154	
	5	0.274	1603753	1.709E-07	1.276	
Funseq2	1	0.276	4208678	6.556E-08	0.490	0.154
	2	0.362	2483215	1.456E-07	1.087	
	3	0.299	2311991	1.292E-07	0.965	
	4	0.260	1861326	1.397E-07	1.043	
CADD	1	0.356	2563253	1.390E-07	1.038	0.066
	2	0.373	2446753	1.526E-07	1.139	
	3	0.284	2310764	1.229E-07	0.918	
	4	0.349	1905380	1.833E-07	1.369	
	5	0.294	1752877	1.677E-07	1.252	
RegulomeDB	1	0.281	3083174	9.120E-08	0.681	3.419
	2	0.368	2111757	1.744E-07	1.302	
	3	*0.457*	*556547*	*8.208E-07*	*6.130*	
	4	*0.423*	*136034*	*3.106E-06*	*23.196*	
	5	*0.343*	*187380*	*1.829E-06*	*13.662*	
Funseq	1	0.357	4990794	7.161E-08	0.535	3.162
	2	0.382	1080633	3.534E-07	2.639	
	3	0.301	228382	1.319E-06	9.854	
	4	*0.051*	*29950*	*1.686E-06*	*12.595*	
	5	*0.000*	*5027*	*1.989E-10*	*0.001*	
	6	*0.008*	*280*	*2.986E-05*	*223.008*	
GenoSkyline (blood)	1	0.354	9702179	3.649E-08	0.273	1.276
	2	*0.173*	*245856*	*7.044E-07*	*5.261*	
	3	*0.089*	*120694*	*7.349E-07*	*5.488*	
	4	*0.221*	*910298*	*2.432E-07*	*1.817*	

(C) Body mass index (BMI)

Annotation	value	h^2 (RV)	#SNV	h^2/#SNV	Normalized (by GERP++=1)	Regression Slopes
GERP++	1	0.126	2610225	4.838E-08	1.000	0.231
	2	0.102	2432456	4.210E-08	0.870	
	3	0.070	1878023	3.745E-08	0.774	
	4	0.182	1781008	1.023E-07	2.115	
	5	0.119	1603753	7.418E-08	1.533	
Funseq2	1	0.163	4208678	3.875E-08	0.801	-0.033
	2	0.158	2483215	6.346E-08	1.312	
	3	0.000	2311991	4.325E-13	0.000	
	4	0.102	1861326	5.458E-08	1.128	
CADD	1	0.103	2563253	4.012E-08	0.829	0.154
	2	0.141	2446753	5.779E-08	1.194	
	3	0.095	2310764	4.110E-08	0.849	
	4	0.138	1905380	7.239E-08	1.496	
	5	0.123	1752877	7.004E-08	1.448	
RegulomeDB	1	0.102	3083174	3.310E-08	0.684	1.633
	2	0.156	2111757	7.404E-08	1.530	
	3	*0.060*	*556547*	*1.075E-07*	*2.223*	
	4	*0.171*	*136034*	*1.255E-06*	*25.944*	
	5	*0.000*	*187380*	*5.337E-12*	*0.000*	
Funseq	1	0.126	4990794	2.516E-08	0.520	5.423
	2	0.123	1080633	1.139E-07	2.354	
	3	0.000	228382	4.379E-12	0.000	
	4	*0.000*	*29950*	*3.339E-11*	*0.001*	
	5	*0.000*	*5027*	*1.989E-10*	*0.004*	
	6	*0.038*	*280*	*1.363E-04*	*2816.763*	
GenoSkyline (blood)	1	0.129	9702179	1.328E-08	0.274	0.940
	2	*0.021*	*245856*	*8.593E-08*	*1.776*	
	3	*0.058*	*120694*	*4.840E-07*	*10.004*	
	4	*0.054*	*910298*	*5.914E-08*	*1.222*	

(D) Systolic blood pressure (SBP)

Annotation	value	h^2 (RV)	#SNV	h^2/#SNV	Normalized (by GERP++=1)	Regression Slopes
GERP++	1	0.256	2610225	9.804E-08	1.000	0.263
	2	0.327	2432456	1.343E-07	1.370	
	3	0.273	1878023	1.456E-07	1.485	
	4	0.377	1781008	2.117E-07	2.159	
	5	0.302	1603753	1.884E-07	1.922	
Funseq2	1	0.337	4208678	8.015E-08	0.818	0.198
	2	0.224	2483215	9.010E-08	0.919	
	3	0.252	2311991	1.089E-07	1.111	
	4	0.258	1861326	1.386E-07	1.413	
CADD	1	0.294	2563253	1.146E-07	1.169	0.165
	2	0.360	2446753	1.470E-07	1.499	
	3	0.256	2310764	1.108E-07	1.130	
	4	0.372	1905380	1.954E-07	1.993	
	5	0.300	1752877	1.713E-07	1.748	
RegulomeDB	1	0.358	3083174	1.161E-07	1.184	2.840
	2	0.279	2111757	1.320E-07	1.346	
	3	*0.151*	*556547*	*2.716E-07*	*2.770*	
	4	*0.285*	*136034*	*2.098E-06*	*21.400*	
	5	*0.355*	*187380*	*1.895E-06*	*19.333*	
Funseq	1	0.328	4990794	6.575E-08	0.671	14.050
	2	0.328	1080633	3.031E-07	3.092	
	3	0.420	228382	1.837E-06	18.736	
	4	*0.159*	*29950*	*5.318E-06*	*54.243*	
	5	*0.028*	*5027*	*5.485E-06*	*55.948*	
	6	*0.000*	*280*	*3.571E-09*	*0.036*	
GenoSkyline (blood)	1	0.329	9702179	3.387E-08	0.345	1.349
	2	*0.066*	*245856*	*2.668E-07*	*2.721*	
	3	*0.055*	*120694*	*4.587E-07*	*4.679*	
	4	*0.260*	*910298*	*2.861E-07*	*2.918*	
