# Supplementary material for: FunSPU: A versatile and adaptive multiple functional annotation-based association test of whole-genome sequencing data
Source: PLoS Genet. 2019 Apr 29;15(4):e1008081. doi: 10.1371/journal.pgen.1008081 (PMC6508749; doi:10.1371/journal.pgen.1008081)
Supplement: S3 Table — (PDF) [file pgen.1008081.s015.pdf]

Supplemental Table S3. Summary and comparison of the proposed tests.

Test	Allow multiple functional annotations as weights?	Way to combine multiple annotations/weights	Comment	recommended for genome-wide scan of WGS data
aSPU(w)*	No	N/A	an omnibus RV association test that covers the burden, SKAT, minP tests and beyond	Yes
aSPU(w)_minP	Yes	Minimum p-value of individual annotation-weighted aSPU	Closely related to the FST test (He et al, AJHG 2017)	Yes
aSPU(w)_Fisher	Yes	Fisher's meta-analysis approach to combine p-values of individual annotation-weighted aSPU	Assuming all annotations are roughly equally informative; often less powerful than aSPU_minP	No
FunSPU(w)	Yes	two-layers of gamma parameters to control each variant's and annotation's contribution to the overall test statistic	more general than aSPU_minP and aSPU_Fisher; the main proposal here	Yes
wtFunSPU(w)	Yes	FunSPU with a data-derived global weight for each annotation	When the global weighting is consistent with the relative informativeness of multiple annotations for a given locus, power gain is expected; otherwise, power loss can occur compared to FunSPU	Yes

*: (w) indicates inverse-variance weighted score function in the SPU framework; variants with lower MAF are up-weighted.
