# Supplementary material for: FunSPU: A versatile and adaptive multiple functional annotation-based association test of whole-genome sequencing data
Source: PLoS Genet. 2019 Apr 29;15(4):e1008081. doi: 10.1371/journal.pgen.1008081 (PMC6508749; doi:10.1371/journal.pgen.1008081)
Supplement: S4 Table — (PDF) [file pgen.1008081.s016.pdf]

Supplemental Table S4. List of heritability of common variants (CVs; MAF>5%) by each category of functional annotation (TWINSUK WGS cohort) estimated by LD score regression. The *italicized* categories of each annotation were merged as one category. The corresponding phenotypes: (A) HDL, (B) LDL

(A) HDL

Annota-tion	value	h^2 (CV)	Proportion of SNPs	h^2/ proportion of SNPs	Normalized (by GERP++=1)	Regression Slopes (common variants by LD score regression)	Regression Slopes (TWINSUK, rare variants by GCTA)
GERP++	1	0.247	0.206	1.202	1.000	0.597	0.204
	2	-0.025	0.197	-0.128	-0.106		
	3	-0.055	0.153	-0.356	-0.296		
	4	0.442	0.138	3.194	2.657		
	5	0.355	0.113	3.131	2.605		
Funseq2	1	0.076	0.352	0.217	0.181	0.890	0.177
	2	0.139	0.200	0.695	0.578		
	3	0.210	0.177	1.186	0.987		
	4	0.478	0.132	3.618	3.010		
CADD	1	0.249	0.221	1.126	0.937	0.506	0.238
	2	0.034	0.213	0.159	0.132		
	3	0.116	0.187	0.622	0.518		
	4	0.151	0.138	1.096	0.912		
	5	0.392	0.106	3.698	3.076		
RegulomeDB	1	-0.166	0.254	-0.655	-0.545	2.833	2.502
	2	-0.017	0.158	-0.111	-0.092		
	3,4,5	0.617	0.069	8.987	7.477		
Funseq	1	0.093	0.170	0.547	0.455	5.329	15.170
	2	0.302	0.073	4.155	3.457		
	3	0.041	0.013	3.255	2.708		
	4,5,6	0.054	0.002	26.359	21.929		
Geno-Skyline (blood)	1	0.564	0.775	0.728	0.606	1.422	5.359
	2,3,4	0.377	0.091	4.146	3.449		

(B) LDL

Annotation	value	h^2 (CV)	Proportion of SNPs	h^2/ proportion of SNPs	Normalized (by GERP++=1)	Regression Slopes (common variants by LD score regression)	Regression Slopes (TWINSUK, rare variants by GCTA)
GERP++	1	0.239	0.206	1.161	1	0.0589	0.046
	2	0.344	0.197	1.745	1.503		
	3	0.045	0.153	0.293	0.253		
	4	0.232	0.138	1.679	1.446		
	5	0.174	0.113	1.537	1.323		
Funseq2	1	0.041	0.352	0.118	0.101	0.9571	0.154
	2	0.159	0.2	0.796	0.686		
	3	0.249	0.177	1.411	1.215		
	4	0.478	0.132	3.616	3.115		
CADD	1	0.195	0.221	0.882	0.76	0.5924	0.066
	2	0.104	0.213	0.487	0.42		
	3	0.127	0.187	0.677	0.583		
	4	0.126	0.138	0.915	0.788		
	5	0.435	0.106	4.108	3.538		
RegulomeDB	1	-0.015	0.254	-0.06	-0.052	2.7101	3.419
	2	0.181	0.158	1.146	0.987		
	3,4,5	0.617	0.069	8.99	7.744		
Funseq	1	-0.176	0.17	-1.037	-0.893	6.516	3.162
	2	0.562	0.073	7.743	6.67		
	3	0.008	0.013	0.643	0.554		
	4,5,6	0.063	0.002	31.121	26.806		
Geno-Skyline (blood)	1	0.615	*0.775*	0.086	0.684	1.416	1.276
	2,3,4	0.371	*0.091*	0.773	3.516		
